# TRIM28 promotes the escape of gastric cancer cells from immune surveillance by increasing PD-L1 abundance

**DOI:** 10.1038/s41392-023-01450-3

**Published:** 2023-06-26

**Authors:** Xiaoxiao Ma, Shuqin Jia, Gangjian Wang, Min Liang, Ting Guo, Hong Du, Sisi Li, Xiaomei Li, Longtao Huangfu, Jianping Guo, Xiaofang Xing, Jiafu Ji

**Affiliations:** 1 Department of Gastrointestinal Cancer Translational Research, Key Laboratory of Carcinogenesis and Translational Research (Ministry of Education), Peking University Cancer Hospital & Institute, Beijing, China; 2 Department of Molecular Diagnostics, Key Laboratory of Carcinogenesis and Translational Research (Ministry of Education), Peking University Cancer Hospital & Institute, Beijing, China; 3 Institute of Precision Medicine, the First Affiliated Hospital, Sun Yat-Sen University, Guangzhou, Guangdong 510275 China; 4 Department of Gastrointestinal Surgery, Key Laboratory of Carcinogenesis and Translational Research (Ministry of Education), Peking University Cancer Hospital & Institute, Beijing, China

**Keywords:** Tumour immunology, Immunotherapy

## Abstract

Immune checkpoint blockade (ICB) offers a new opportunity for treatment for gastric cancer (G.C.). Understanding the upstream regulation of immune checkpoints is crucial to further improve the efficacy of ICB therapy. Herein, using the CRISPR-Cas9-based genome-wide screening, we identified TRIM28 as one of the most significant regulators of PD-L1, a checkpoint protein, in G.C. cells. Mechanistically, TRIM28 directly binds to and stabilizes PD-L1 by inhibiting PD-L1 ubiquitination and promoting PD-L1 SUMOylation. Furthermore, TRIM28 facilitates K63 polyubiquitination of TBK1, activating TBK1-IRF1 and TBK1-mTOR pathways, resulting in enhanced *PD-L1* transcription. It was found that TRIM28 was positively correlated with PD-L1 in G.C. cells. Moreover, high TRIM28 expression suggests poor survival in a cohort of 466 patients with G.C., and this observation is consistent while analyzing data from publicly available databases. Ectopic TRIM28 expression facilitated tumor growth, increased PD-L1 expression, and suppressed T cell activation in mice. Administration of the PD-L1 or TBK1 inhibitor significantly alleviated the TRIM28-induced tumor progression. Furthermore, combining the TBK1 inhibitor with CTLA4 immune checkpoint blockade has synergistic effects on G.C., and provides a novel strategy for G.C. therapy.

## Introduction

Although the incidence rate of gastric cancer (G.C.) and the associated mortality continue to decrease, 1,089,103 individuals were diagnosed with G.C. worldwide in 2020; approximately 768,793 died, making it the third leading cause of cancer-related deaths in that year. Approximately 44% (478,508) of these cases were reported from China.^1,2^ Identifying immune checkpoints (I.C.) and developing I.C. inhibitors (ICI) have revolutionized cancer therapy. Immunotherapy that blocks the PD-1/PD-L1 pathway has recently been employed for G.C. treatment.^3^ Pembrolizumab (Keytruda), a humanized antibody, has been approved for immunotherapy in patients with recurrent locally advanced or metastatic gastric or gastroesophageal junction (GEJ) adenocarcinoma expressing PD-L1.^4^ Accumulating evidence reveals that PD-L1 is an essential biomarker for determining the efficacy of immunotherapy.^5–8^ Therefore, understanding the molecular mechanism regulating the expression and/or stability of PD-L1 in G.C. cells will provide a molecular evidence for improving the clinical response of immunotherapies.

Whole-genome CRISPR-Cas9 screens are a powerful tool for identifying key genes or biomarkers mediating drug resistance.^9–11^ A better understanding of these genes will help develop novel combination therapies.^12^ Several PD-L1 regulators, including CMTM6 and CMTM4, have been identified via CRISPR screens, which potently facilitate combination immunotherapies for cancer;^13–15^ however, such screening approach has not been utilized in G.C. MAGeCK is a comprehensive workflow that can be used for CRISPR screening data analyses.^16,17^

Tripartite motif-containing 28 (TRIM28) is a pivotal regulator of DNA damage response by recruiting DNA damage response factors at the site of DNA strand breaks.^18^ TRIM28 contains an amino (N) terminus TRIM structure and a C-terminal PHD-Bromo dual epigenetic reader domain, acting as a canonical RING-type E3 ubiquitin ligase.^15,19,20^ Besides acting as a canonical RING-type E3 ubiquitin ligase, TRIM28 promotes SUMOylation of target proteins by acting as an E3 SUMO ligase under certain conditions.^20–22^ It has been reported that TRIM28 binds to and protects NLRP3 from proteasomal degradation by inhibiting NLRP3 ubiquitination and promoting NLRP3 SUMOylation.^21^ Ubiquitination and SUMOylation are two essential components of the ubiquitination-proteasome system. The crosstalk between these two processes plays fundamental roles in protein homeostasis and signal transduction, influencing carcinogenesis.

Herein, we performed a FACS-based genome-wide CRISPR-Cas9 screening in N87 gastric cancer cell line. The abundance of sgRNAs was analyzed and compared between PD-L1-low and PD-L1-high subpopulations using MAGeCK. Our screen identified TRIM28, also named as TIF1β and KAP1, as one of the top-ranking putative regulators of PD-L1. We performed several biochemical analyses to elucidate the effect of TRIM28 on PD-L1 stability and expression in G.C. cells. To investigate the effect of *TRIM28* depletion or overexpression on global gene expression in G.C. cells, RNA-seq was performed. Using the integrated bioinformatics analyses, mouse models, and clinical observations, we tried to uncover the effect of TRIM28-mediated PD-L1 upregulation on tumor growth and anti-tumor immunity. Together, our study not only reveal a novel mechanism that regulates PD-L1 expression and stability but also highlight a potential strategy of using TBK1 inhibitors in combination with CTLA4 immune checkpoint blockade to treat G.C. efficiently.

## Results

### Genome-wide CRISPR/Cas9-based screening to identify genes regulating PD-L1 expression in gastric cancer

**Fig. 1:**
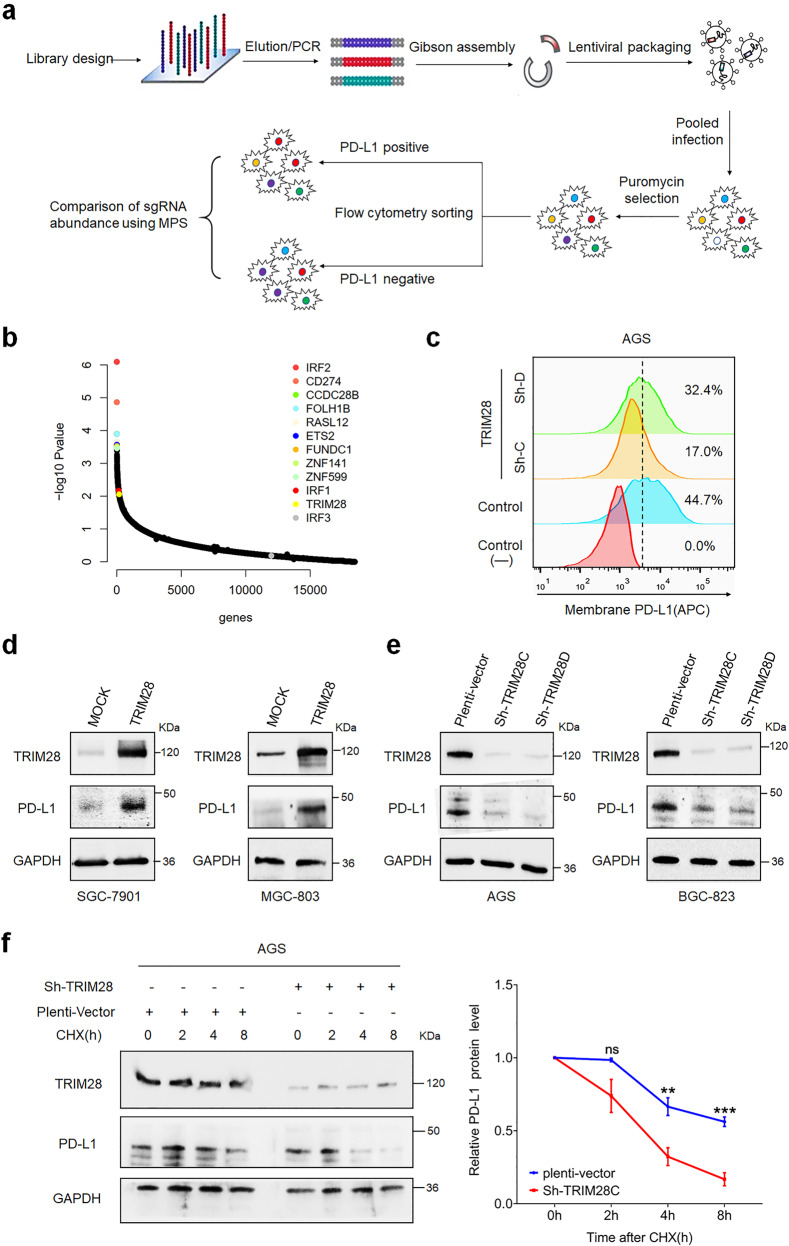
TRIM28 stabilizes PD-L1 in G.C. cells. **a** Genome-wide CRISPR screen identified genes that possibly regulate PD-L1 expression in N87 gastric cancer cells. **b** MAGeCK-VISPR algorithm was used to assess the sgRNA abundance in PD-L1 positive and negative cells by calculating the beta score for each gene. **c** Flow-cytometric analysis of the cell surface PD-L1 expression in AGS cells stably transduced with lentiviruses containing the empty vectors, sh-TRIM28C, or sh-TRIM28D. **d** SGC-7901 and MGC-803 cells were transfected with TRIM28 encoding constructs, and western blotting was performed to detect TRIM28 and PD-L1 protein levels. **e** Western blot analysis of TRIM28 and PD-L1 protein levels in AGS and BGC-823 cells stably transduced with lentiviruses containing the empty vectors, sh-TRIM28C, or sh-TRIM28D. **f** AGS cells were transduced with lentiviruses containing the empty vectors or sh-TRIM28C and incubated with CHX for 0, 2, 4, and 8 h. Following this, the half-life of PD-L1 protein in different groups was evaluated. Bar = means ± SD; *n* = 3; ns, no significance; *P < 0.05; **P < 0.01; ***P < 0.001

To find genes regulating PD-L1 expression in G.C, we performed genome-wide CRISPR-Cas9-based screening in N87 gastric cancer cell line. Cells were infected with lentiviruses containing the guide RNA (gRNA), and selection was performed with puromycin. The transduced cells were sorted based on PD-L1 expression and subjected to deep sequencing analysis (Fig. 1a). Subsequently, genes that negatively or positively regulate PD-L1 expression were scored using the MAGeCK algorithm by calculating the beta score for each gene.^16,17^ In our results, genes that are the known regulators of PD-L1 expression, such as CD274, IRF2, STAT3, and IRF1, were negatively selected (Fig. 1b), confirming the efficiency and precision of our screening system. Further, TRIM28 was on the top of the 300 positively selected genes regulating PD-L1 stability (Fig. 1b). To verify this observation, we performed CRISPR-Cas9-mediated *TRIM28* knockout, and results showed that loss of *TRIM28* markedly decreased PD-L1 expression in G.C. cells (Fig. 1c).

### TRIM28 stabilizes PD-L1 in G.C. cells

Next, we investigated the relevance between TRIM28 and PD-L1 expression. First, we detected the basic expression levels of TRIM28 in multiple G.C. cell lines (Supplementary Fig. S1a). We modulated TRIM28 expression in different G.C. cell lines. Results showed that TRIM28 overexpression significantly increased PD-L1 protein levels in SGC-7901 and MGC-803 cells (Fig. 1d), while *TRIM28* depletion reduced PD-L1 expression in AGS and BGC-823 cells (Fig. 1e), suggesting that TRIM28 positively regulates PD-L1 expression in G.C. cells. It has been reported that PD-L1 readily undergoes post-translational modifications; thus, we further investigated whether TRIM28 directly regulates PD-L1 protein stability. Results showed that *TRIM28* depletion significantly shortened the half-life of PD-L1 (Fig. 1f), indicating that TRIM28 stabilizes PD-L1 protein in G.C. cells. Intriguingly, we observed that TRIM28 could also upregulate *PD-L1* mRNA levels (Supplementary Fig. S1b), suggesting that TRIM28 likely has regulatory effects on both the mRNA and protein levels of PD-L1.

### TRIM28 specifically interacts with PD-L1

**Fig. 2:**
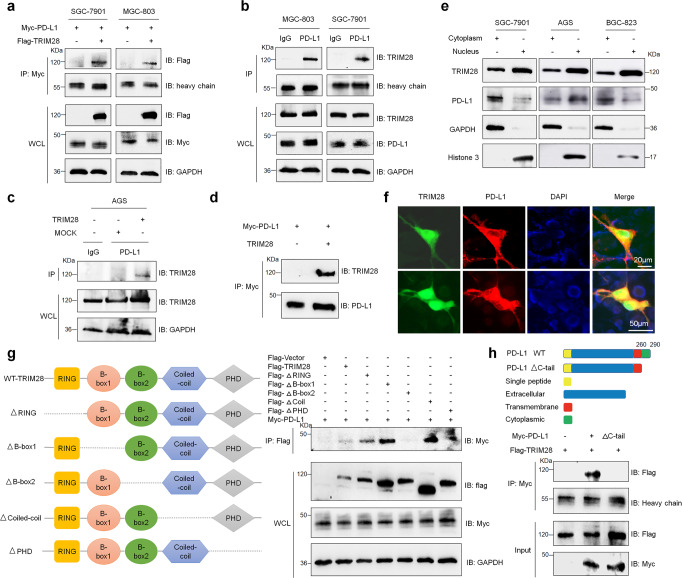
TRIM28 specifically interacts with PD-L1. **a**–**c** Co-IP analysis to confirm the binding of TRIM28 with PD-L1 in G.C. cells. **d** Western blot analysis showing direct interaction between TRIM28 and Myc-PD-L1 proteins produced using the in vitro transcription and translation systems. **e** Western blot analysis showing the expression of TRIM28 and PD-L1 in the cytoplasmic and nuclear fractions of G.C. cells. **f** Confocal microscopy analysis showing the colocalization of TRIM28 (Green) and PD-L1 (Red) in MGC-803 cells. **g** Schematic representation of TRIM28 full-length and truncated mutants. Co-IP analysis for investigating the interaction between PD-L1 and TRIM28 full-length or TRIM28 truncated mutants in G.C. cells. **h** Schematic representation of PD-L1 full-length and truncated mutants. Co-IP analysis of the interaction between TRIM28 and PD-L1 full-length or ΔC-tail in G.C. cells

Since TRIM28 not only targets proteins for ubiquitination and degradation, but also promotes SUMOylation, we investigated whether TRIM28 regulates PD-L1 stability via SUMOylation or ubiquitination in G.C. cells. First, we detected whether TRIM28 interacts with PD-L1 by using co-immunoprecipitation (co-IP) assays. Results suggested that both ectopically expressed (Fig. 2a) and endogenous TRIM28 (Fig. 2b, c) interacts with PD-L1. Next, using the in vitro transcription and translation system to produce the recombinant proteins, direct interaction between TRIM28 and PD-L1 was observed in vitro (Fig. 2d). Consistently, laser confocal-based detection and subcellular fractionation analyses showed that TRIM28 and PD-L1 colocalized in the nucleus and cytoplasm of G.C. cells (Fig. 2e, f), besides, confocal microscopy analysis in tissue microarray also showed consistent results (Supplementary Fig. S1c), implying that TRIM28 possibly affects both cytoplasmic as well as nuclear functions of PD-L1 in G.C. cells. Since TRIM28 contains multiple domains, it interacts with different partners via distinct domains. To find the TRIM28 domains necessary for TRIM28-PD-L1 interaction, we generated truncated TRIM28 and PD-L1 peptides. Results showed that the B-box2 domain of TRIM28 was necessary for its interaction with PD-L1 (Fig. 2g). Moreover, the C-tail plasma domain of PD-L1 was found to be crucial for its binding with TRIM28 (Fig. 2h). These results together demonstrate that TRIM28 directly interacts with the C-tail plasma domain of PD-L1.

### TRIM28 protects PD-L1 from proteasome-mediated degradation by promoting PD-L1 SUMOylation

**Fig. 3:**
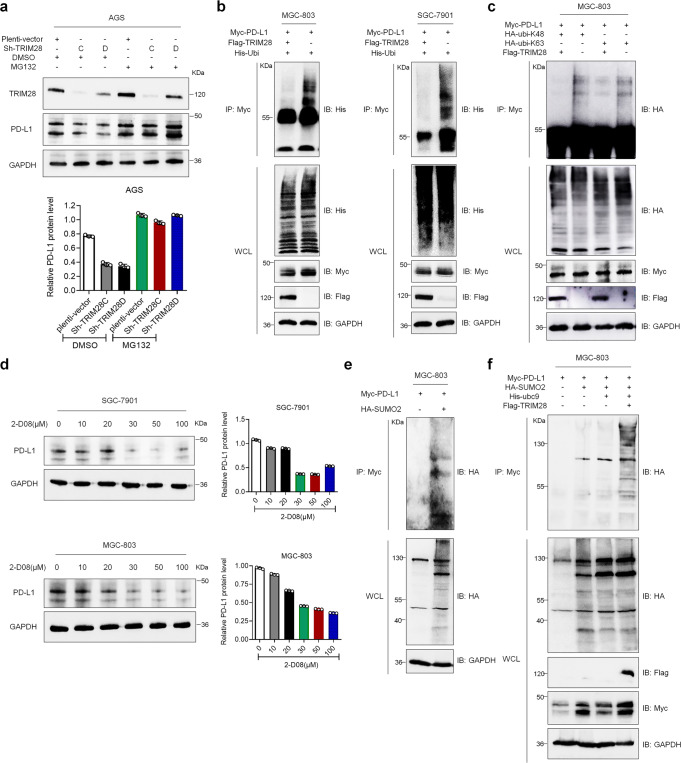
TRIM28 protects PD-L1 from proteasome-mediated degradation and promotes SUMOylation of PD-L1. **a** Western blot analysis of TRIM28 and PD-L1 protein levels in AGS cells stably transduced with lentiviruses containing the empty vectors, sh-TRIM28C, or sh-TRIM28D following treatment with DMSO or MG132 (10 μM). **b** Co-IP analysis of ubiquitinated PD-L1 in MGC-803 and SGC-7901 cells co-transfected with constructs encoding Myc-PD-L1, Flag-TRIM28, and His-ubi. **c** Co-IP analysis of ubiquitinated PD-L1 in MGC-803 cells transfected with the indicated constructs. **d** Western blot analysis of PD-L1 protein levels in SGC-7901 or MGC-803 cells stimulated with different concentrations of 2-D08 for 24 h. **e**, **f** SUMOylation assays in the lysates from MGC-803 cells transfected with indicated constructs

The autophagy-lysosome and ubiquitin-proteasome systems are two major protein degradation pathways. To elucidate how TRIM28 regulates PD-L1 stability, we determined to treat *TRIM28*-depleted G.C. cells with the proteasome inhibitor MG132. Results displayed that MG132 treatment blocked PD-L1 protein degradation in *TRIM28*-depleted cells (Fig. 3a), indicating that TRIM28 selectively inhibits the ubiquitin-proteasome-mediated degradation of PD-L1. Ubiquitination, especially K48 ubiquitination, is essential for the degradation of proteins through the ubiquitin-proteasome system. Thus, we investigated whether TRIM28 influences PD-L1 ubiquitination. Results displayed that the levels of polyubiquitinated PD-L1 were markedly reduced in TRIM28 overexpressing MGC-803 and SGC-7901 cells (Fig. 3b). Moreover, TRIM28 overexpression inhibited both K48-linked and K63-linked ubiquitination of the endogenous PD-L1 (Fig. 3c). These results indicate that although TRIM28 is an E3 ubiquitin ligase, it protects PD-L1 from proteasome-mediated degradation by preventing PD-L1 polyubiquitination.

Since previous studies showed that TRIM28 could promote SUMOylation of the target proteins to prevent their ubiquitination and subsequent degradation,^21–23^ we investigated whether TRIM28 promotes PD-L1 SUMOylation. G.C. cells were treated with 2-D08, an inhibitor of protein SUMOylation, and results showed that PD-L1 expression was reduced in a dose-dependent manner, suggesting a potential role of SUMOylation in PD-L1 stabilization (Fig. 3d). Furthermore, results showed that PD-L1 could be post-translationally modified by SUMO2 (Fig. 3e), and ectopic expression of TRIM28 enhanced SUMO2 modification of PD-L1 in MGC-803 cells (Fig. 3f). Collectively, these results indicate that TRIM28 promotes PD-L1 SUMOylation, prevents PD-L1 polyubiquitination, and stabilizes the PD-L1 protein in G.C. cells.

### TRIM28 enhances *PD-L1* transcription by activating the TBK1-IRF1 and TBK1-mTOR pathways

**Fig. 4:**
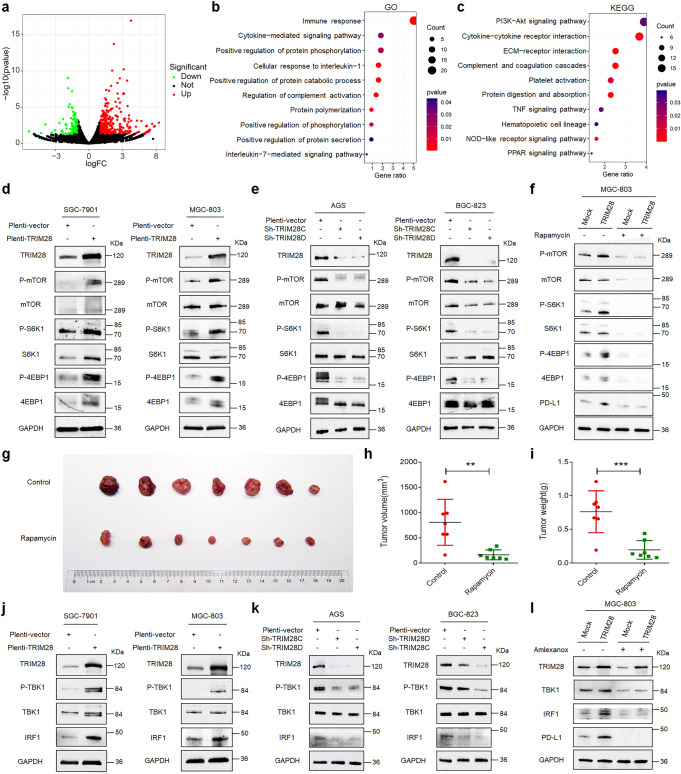
TRIM28 post-translationally modifies PD-L1 via TBK1-IRF1 and TBK1-mTOR pathways. **a**–**c** RNA-sequencing analysis of SGC-7901 cells transfected with TRIM28 or control plasmids. **d** Western blot analysis of the mTOR signaling proteins in SGC-7901 and MGC-803 cells transduced with indicated lentivirus. **e** Western blot analysis of the mTOR signaling proteins in AGS and BGC-823 cells transduced with indicated lentivirus. **f** Analysis of the mTORC1-PD-L1 signaling proteins by western blot in MGC-803 cells transduced with lentiviral particles containing indicated constructs following rapamycin treatment. **g** TRIM28 expressing MFC cells were laterally injected into the abdomen of 615 mice treated with/without the mTOR inhibitor rapamycin. **h**, **i** The volume and weight of the formed tumors in different groups. **j** SGC-7901 and MGC-803 cells infected with the indicated lentivirus and subjected to western blot analysis. **k** AGS and BGC-823 cells infected with the indicated lentivirus and subjected to western blot analysis. **l** AGS cells infected with the indicated lentivirus were treated with amlexanox and subjected to western blot analysis. *P < 0.05; **P < 0.01; ***P < 0.001

In order to explore the effect of TRIM28 in regulating *PD-L1* transcription, we analyzed the transcriptome of TRIM28 overexpressing G.C. cells by RNA-seq (Fig. 4a–c). Gene Ontology (GO) analysis revealed that TRIM28 regulates genes participated in the immune response, cytokine-mediated signaling pathway, and phosphorylated modifications (Fig. 4b). Furthermore, KEGG pathway analysis revealed that TRIM28 regulates several cancer-associated pathways such as the PI3K-AKT, TNF, and PPAR pathways (Fig. 4c). Our results revealed that the PI3K-AKT-mTOR signaling pathway was significantly activated in cells overexpressing TRIM28, as indicated by increased levels of p-4E-BP1, p-S6K1, and p-mTOR (Fig. 4d). In contrast, *TRIM28* knockdown markedly diminished the mTOR pathway activity (Fig. 4e). To investigate whether mTOR plays a role in TRIM28-mediated regulation of PD-L1 expression, we used the mTOR-specific inhibitor rapamycin. Results showed that rapamycin prevented the TRIM28-mediated increase in PD-L1 expression (Fig. 4f), suggesting that mTOR signaling partially mediates TRIM28 functions to induce PD-L1 expression. In addition, rapamycin treatment markedly reduced the tumor burden in immuno-proficient mice bearing TRIM28-expressing tumors (Fig. 4g–i).

Interestingly, we also observed that TRIM28 overexpression significantly increased the levels of p-TBK1 and IRF1 (Fig. 4j), key proteins that play critical roles in the innate immune response. Consistent with previous reports that IRF1 directly binds to the *CD274* (PD-L1) gene promoter and increase *PD-L1* expression,^24–27^ *TRIM28* depletion significantly attenuated the TBK1-IRF1 signaling by decreasing TBK1 phosphorylation and IRF1 expression (Fig. 4k). In support of this finding, the TBK1 inhibitor amlexanox markedly decreased the levels of PD-L1, p-TBK1, and IRF1, suggesting that PD-L1 is downstream of the TBK1-IRF1 signaling (Fig. 4l). Consistently, TBK1 inhibitors reduced the expression of PD-L1 in TRIM28 overexpressing cells (Fig. 4l), indicating that TBK1 plays a vital role in TRIM28-mediated regulation of PD-L1 expression.

### TRIM28 interacts with and activates TBK1 by enhancing its K63-linked polyubiquitination

**Fig. 5:**
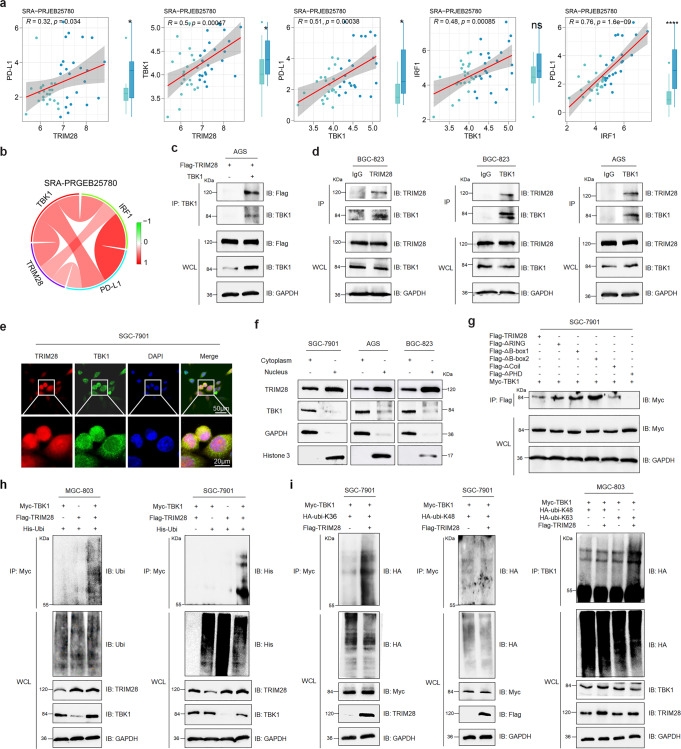
TRIM28 interacts with and activates TBK1 by promoting its K63-linked ubiquitination. **a**, **b** Correlation between TRIM28 expression and the expression of PD-L1, TBK1, and IRF1 analyzed using the SRA-PRJEB25780 dataset. **c**, **d** Co-IP analysis for evaluating the interaction between TRIM28 and TBK1 in G.C. cells. **e** Confocal microscopy analysis showing colocalization of TRIM28 (Red) and TBK1 (Green) in SGC-7901 cells. **f** Western blot analysis of the cytoplasmic and nuclear fractions derived from G.C. cells. **g** Co-IP analysis of the interaction between TBK1 and TRIM28 full-length or TRIM28 truncated mutants in G.C. cells. **h** Co-IP analysis of ubiquitinated TBK1 in MGC-803 and SGC-7901 cells transfected with indicated constructs. **i** Co-IP analysis of ubiquitinated TBK1 in SGC-7901 and MGC-803 cells transfected with indicated constructs

Our analysis of the Sequence Read Archive (SRA), Gene Expression Omnibus (GEO), and Asian Cancer Research Group (ACRG) databases showed that TRIM28 is positively associated with PD-L1, TBK1, and IRF1 at the transcriptional level (Fig. 5a, b and Supplementary Fig. S2). Therefore, we investigated whether the E3 ubiquitin ligase TRIM28 is involved in regulating the TBK1-IRF1-PD-L1 pathway. Results showed that TBK1 interacted with TRIM28 (Fig. 5c, d). The results of confocal and subcellular fractionation assays displayed that TRIM28 colocalized with TBK1 in the cytoplasm of G.C. cells (Fig. 5e, f); besides, confocal microscopy analysis in tissue microarray also showed consistent results (Supplementary Fig. S3a), and the PHD domain of TRIM28 was critical for its interaction with TBK1 (Fig. 5g).

Next, we investigated whether TRIM28 is involved in regulating TBK1 activity. Results showed that WT, but not Ring domain-deleted (ΔRing)-TRIM28 promoted TBK1 ubiquitination (Fig. 5h and Supplementary Fig. S3b). Specifically, TRIM28 promoted the K63 but no K48 polyubiquitination of TBK1 (Fig. 5i). Taken together, these data demonstrate that TRIM28 activates TBK1 by promoting its K63-linked ubiquitination.

### TRIM28-mediated PD-L1 upregulation promotes tumor growth and inhibits anti-tumor immunity

**Fig. 6:**
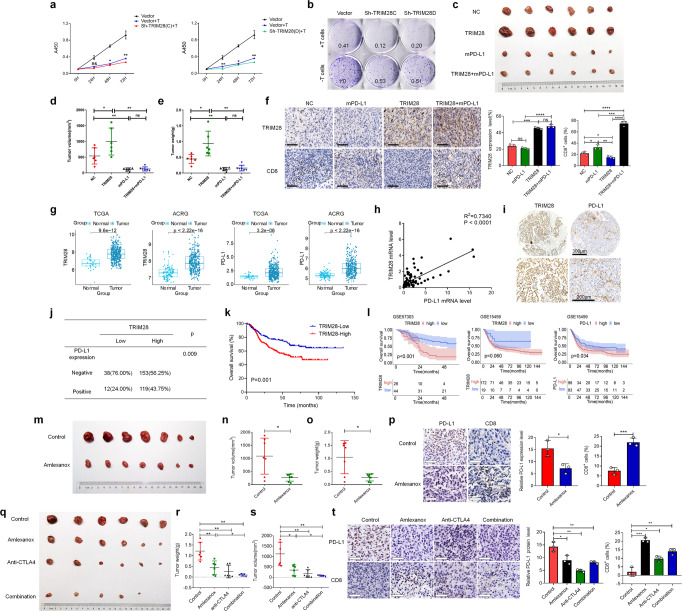
TRIM28-mediated PD-L1 upregulation promotes G.C. tumor growth and inhibits antitumor immunity. TRIM28-mediated PD-L1 upregulation promotes G.C. tumor growth and inhibits antitumor immunity. **a**, **b** Assays evaluating T cell-mediated tumor cell death. **c** WT and TRIM28 overexpressing MFC cells were laterally injected into the abdomen of 615 mice treated with either anti-PD-L1 antibodies or IgG isotype control and randomly assigned to different groups as indicated. Representative images of the 615 mouse tumors. **d**, **e** The volume and weight of the formed tumors. **f** Immunohistochemistry analysis showing TRIM28 expression, and the number of infiltrated CD8^+^ T cells in tumors derived from the WT or TRIM28 overexpressing MFC cells. Scale bar, 50 μm. **g** Analysis of *TRIM28* and *PD-L1* mRNA expression in G.C. patients using ACRG and TCGA databases. **h** Correlation between *TRIM28* and *PD-L1* mRNA levels in G.C. tissues from our cohort. **i**, Representative immunohistochemical staining of G.C. tumors showing TRIM28 and PD-L1 expression. **j** Correlation between TRIM28 and PD-L1 protein levels in G.C. tissues from our cohort. **k** Kaplan-Meier survival analysis based on TRIM28 expression in G.C. patients from our cohort. **l** Kaplan-Meier survival analysis based on TRIM28 expression in G.C. patients from the GEO database. **m** TRIM28 expressing MFC cells were laterally injected into the abdomen of 615 mice treated with or without the TBK1 inhibitor amlexanox. **n**, **o** The volume and weight of the formed tumors in different groups. **p** Immunohistochemistry analysis of PD-L1 expression and the number of infiltrated CD8^+^ T cells in tumors from different groups. Scale bar, 50 μm. **q** Mice carrying tumors derived from MFC cells were treated with either the TBK1 inhibitor amlexanox or anti-CTLA4 antibodies individually or a combination of both. **r**, **s** The volume and weight of the formed tumors in different groups. **t** Immunohistochemistry analysis of PD-L1 expression and the number of infiltrated CD8^+^ T cells in tumors from different groups. Scale bar, 50 μm. Bar = means ± SD; *n* = 3; ns, no significance; *P < 0.05; **P < 0.01; ***P < 0.001

PD-L1 plays a critical role in blocking the immune response and our results showed that TRIM28 was involved in regulating PD-L1 expression. We observed that *TRIM28* ablation markedly enhanced the T-cell-mediated destruction of gastric cancer cells (Fig. 6a, b). In addition, by analyzing multiple cancer types using the ESTIMATED algorithm, we observed that TRIM28 expression is negatively correlated with CD8^+^ T cell infiltration and positively correlated with myeloid-derived suppressor cells infiltration of the solid tumors, and similar results were observed in our experiments (Supplementary Fig. S4). To explore the potential impact of TRIM28 on tumorigenesis in vivo, we employed a syngeneic G.C. mouse model to validate the importance of TRIM28 in regulating the tumor immune response. We observed that mice bearing tumors derived from cells with ectopic TRIM28 expression exhibited faster tumor progression than the control group (Fig. 6c–e). Furthermore, ectopic expression of TRIM28 led decreased infiltration of CD8^+^ tumor-infiltrating lymphocytes (Fig. 6f). To determine whether TRIM28-mediated increased of PD-L1 expression contributes to the immune evasion of the tumor cells, we blocked PD-L1 expression. Results showed that suppressing PD-L1 expression inhibited the TRIM28-induced tumor growth in the syngeneic mouse model by increasing the CD8^+^ T cell infiltration (Fig. 6c–e).

### TRIM28 expression is positively correlated with PD-L1 expression in G.C. patients

Furtherly, to explore the association of TRIM28 and PD-L1 in G.C., we extracted the data of G.C. patients from the Cancer Genome Atlas (TCGA) and ACRG databases and evaluated the expression of TRIM28 and PD-L1 in the tumor and adjacent normal tissues. Results revealed that TRIM28 and PD-L1 were highly expressed in G.C. compared to normal tissues (Fig. 6g). In addition, pan-cancer analyses showed that TRIM28 was highly expressed in 18 types of tumors based on the TCGA database (Supplementary Fig. S5).

Next, we evaluated the mRNA levels of TRIM28 and PD-L1 in 98 G.C. samples. Results suggest that TRIM28 expression is positively correlated with PD-L1 expression (Fig. 6h). We also analyzed 466 G.C. samples to evaluate TRIM28 protein expression by IHC. Low and high TRIM28 expression was observed in 143 (30.7%) and 323 (69.3%) samples, respectively (Fig. 6h, Table 1). Correlation analysis demonstrated that TRIM28 expression was positively associated with an advanced pTNM stage (Table 1). In addition, PD-L1 expression was analyzed in 322 of the 466 G.C. samples, and results showed that 59.3% of samples were PD-L1 negative while 40.7% were PD-L1 positive. Notably, TRIM28 and PD-L1 expression showed a strong positive correlation (Fig. 6i, j). We also observed that TRIM28 expression is positively correlated with PD-L1, TBK1, and IRF1 levels in various cancers (Supplementary Fig. S6). Interestingly, survival analysis of G.C. patients from our cohort and the GEO dataset showed that the overall survival rates of patients with high expression of TRIM28 was poor (Fig. 6k, l), indicating that TRIM28 could serve as a biomarker for G.C. prognosis.

**Table 1:** Clinicopathological characteristics of TRIM28 expression in GC patients

	TRIM28 expression		*P* value
Number	Low	High	
Age			0.280
≤60	83	170	
>60	60	153	
Gender			0.274
Female	47	90	
Male	96	233	
Tumor location			0.198
Lower	52	98	
Middle	31	62	
Upper	56	147	
Whole	1	0	
Histology			0.176
Adenocarcinoma	123	288	
SRCC^a^	20	31	
Differentiation			0.624
Poor	66	142	
Moderate	61	159	
Well	3	10	
Tumor size			0.122
<5 cm	85	173	
≥5 cm	50	141	
Vascular invasion			0.520
Negative	71	149	
Positive	72	172	
Lauren type			0.018
Diffused	46	72	
Intestinal	70	175	
Mixed	20	73	
pT stage			0.022
1	19	19	
2	22	39	
3	72	199	
4	30	66	
pN stage			0.003
1	61	85	
2	24	60	
3	25	59	
4	33	118	
pM stage			0.234
0	132	291	
1	6	23	
pTNM stage			0.018
1	31	39	
2	48	99	
3	53	153	
4	6	23	
cTNM stage			0.172
1	27	36	
2	54	114	
3	48	114	
4	6	21	

^a^SRCC, signet-ring cell carcinoma

Since tumor mutational burden (TMB) can be used to predict the tumor immune signature,^28^ we performed correlation analysis between TRIM28 expression and TMB. Results showed that TRIM28 expression was positively correlated with TMB in multiple cancer types, indicating that TRIM28 might be a robust biomarker for initiating immunotherapy in multiple cancers (Supplementary Fig. S7a). Similar results were observed when analyzing response of immunotherapy (Supplementary Fig. S7b, c).

### Targeting the TRIM28-TBK1 axis enhances the efficacy of cancer immunotherapy

Since TBK1 mediates the roles of TRIM28 on PD-L1 expression, we employed a syngeneic mouse model implanted with TRIM28 overexpressing MFC cells to assess the therapeutic efficacy of targeting TBK1. Treatment with amlexanox, a selective inhibitor of TBK1, strongly suppressed tumor growth in these mice (Fig. 6m–o). Furthermore, amlexanox significantly reduced the PD-L1 expression and increased the infiltration of CD8^+^ T cells into the tumor in this cancer model (Fig. 6p).

Since the combination of blocking PD1/PD-L1 expression and using anti-CTLA4 antibodies is an efficient immunotherapy approach,^29–31^ and our results displayed that the TBK1 inhibitor could repress PD-L1 expression, we tested the synergistic effect of the TBK1 inhibitor and anti-CTLA4 antibodies on G.C. Results showed that although treatment of the immuno-proficient mice with either amlexanox or anti-CTLA4 antibodies suppressed the G.C. growth significantly (Fig. 6q), the combination of both had a more profound effect as evident by reduced PD-L1 expression and increased CD8^+^ T cell infiltration of the tumor (Fig. 6r–t). Taken together, these results indicate that TRIM28-mediated PD-L1 upregulation is partially dependent on TBK1 and highlight a potential strategy for combining the TRIM28 or TBK1 inhibitor with CTLA4 checkpoint blockade for the effective remedy of G.C (Supplementary Fig. S8).

## Discussion

Immunotherapy, especially targeting immune checkpoints, such as CTLA4 or PD1/PD-L1, has been broadly approved for treating human cancers and exhibits durable clinical benefits.^6,32,33^ Emerging evidence suggests that PD-L1 expression levels in tumor cells might determine the clinical response to PD1/PD-L1 blockade.^34,35^ Hence, it is essential to understand the molecular mechanisms underlying controlling PD-L1 protein expression and stability, especially in G.C. Herein, we employed a FACS-based whole-genome CRISPR-Cas9 screen in G.C. cells to identify genes that control PD-L1 expression. Notably, we identified the E3 ubiquitin and SUMO E3 ligase TRIM28 as a direct or indirect regulator of PD-L1 protein stability. Moreover, results from the in vivo tumor models and human G.C. further validated that TRIM28 mediates the immune response by upregulating PD-L1 expression. The function of TRIM28 in controlling PD-L1 levels is controversial since TRIM28 is a multi-domain protein with multiple enzymatic activities. TRIM28 performs complex biological functions in distinct contexts. For example, it has been reported that the SETDB1-TRIM28 complex inhibited PD-L1 expression in ovarian cancer cells,^15^ which is contrary to the results of our study in G.C. cells. These differences might be due to inherent differences between different cancers. Using the publicly available databases, we investigated the correlation of TRIM28 with PD-L1 expression in various cancer types. Results showed that TRIM28 expression is positively correlated with PD-L1 expression in G.C. Our observation is consistent with that of a previous study demonstrating that verteporfin, a photosensitizing drug, suppresses PD-L1 expression by impairing the STAT1-IRF1-TRIM28 complex,^27^ confirming that TRIM28 positively regulates PD-L1 expression. However, none of the previous studies have investigated the specific role of TRIM28 in regulating PD-L1, especially in the post-translational level. Since PD-L1 is controlled by multiple and complex regulatory networks in different conditions, it is important to investigate the molecular mechanism underlying TRIM28-mediated upregulation of PD-L1, especially for the development of combination strategies. Herein, we focused on the E3 ubiquitin ligase and E3 SUMO ligase activities of TRIM28, rather than its transcriptional co-repressor activity, to investigate the effect of TRIM28 on PD-L1 regulation.

The expression of PD-L1 in tumor cells is crucial for their escape from antitumor immunity.^8^ The regulation of PD-L1 expression has been broadly studied at different levels, including transcriptional, translational, and post-translational levels, in distinct cancer types.^5–8,36^ It has been observed that multiple post-translational modifications, such as glycosylation, ubiquitination, phosphorylation, palmitoylation, and acetylation regulate the expression and functions of PD-L1.^37^ Herein, we demonstrated that TRIM28 positively regulates the mRNA and protein levels of PD-L1. TRIM28 inhibits PD-L1 ubiquitination while promoting PD-L1 SUMOylation to block its proteasome-dependent degradation. Conversely, TRIM28 ubiquitinates and activates TBK1 to regulate the TBK1/IRF1 and mTOR pathways, leading to increased transcription of *PD-L1*. It is well established that TRIM28 could protect various substrates, such as TRIM24, CARM1, and NLRP3 from proteasome-mediated degradation.^18,21,22^ Our results enclose that TRIM28 inhibits PD-L1 ubiquitination and subsequent degradation possibly by enhancing its SUMOylation. It has been reported that TRIM28 promotes the nuclear localization of KOX1, ZNF268, and ZNF300 transcription factors. Our data showed that TRIM28 and PD-L1 colocalize both in the nucleus and the cytoplasm of G.C. cells, suggesting that TRIM28 might also mediate the nuclear localization and functions of PD-L1. Taken together, our results reveal that TRIM28 switches the PD-L1 modification from ubiquitination to SUMOylation to prevent PD-L1 degradation.

Besides post-translational modifications, TRIM28 is also involved in transcriptional repression.^38,39^ Our transcriptome analysis revealed that the PI3K-AKT-mTOR axis partially mediates the effects of TRIM28 on *PD-L1* transcription. Although TBK1-mediated regulation of PD-L1 has been reported in lung cancer,^40^ the role of TBK1 in controlling PD-L1 expression in G.C. remains unclear. Herein, we observed that TBK1 and mTOR inhibitors strongly prevented the TRIM28-induced upregulation of PD-L1 expression and inhibited tumor growth in vivo. SUMOylation inhibitors could also impair the TRIM28-mediated mTOR pathway activation while mildly influencing TBK1 expression (Supplementary Fig. S9). TRIM28 catalyzes the K63-linked polyubiquitination of TBK1, resulting in the activation of its downstream substrates IRF1 and mTOR, ultimately resulting in enhanced PD-L1 expression. This provides the basis for using TBK1 inhibitor to prevent the immune escape of G.C. cells. However, it remains unclear whether TBK1 undergoing SUMOylation will influence TRIM28-mediated its ubiquitination kinase activity.

Due to the significant effects of CTLA4 in regulating T-cell activation and suppressing dendritic cell activity by boosting regulatory T (Treg) cells,^31,41^ combining anti-PD1/PD-L1 immunotherapy with inhibiting CTLA4 is clinically beneficial.^29,31,42,43^ Moreover, since TBK1 mediates the influences of TRIM28 on PD-L1 expression, using TBK1 inhibitors could synergize the therapeutic efficacy of anti-CTLA4 treatment on G.C. Combining anti-CTLA4 antibodies with the TBK1 inhibitor amlexanox markedly diluted the tumor burden in G.C. mouse models, consistent with a previous study in the KrasLA2 mouse model.^40^ Our results provided evidence that on one hand, inhibition of TRIM28/TBK1 pathway decreased PD-L1 level which has been linked with improved response to ICB; on the other hand, it increased the infiltration and activation of T cells in the tumor microenvironment to boost antitumor immunity and further promote tumor eradication. Specific TRIM28 inhibitors could be further developed and validated in combination with anti-CTLA4 therapy to suppress tumor growth efficiently.

In conclusion, our study uncovers a novel role of TRIM28 in regulating PD-L1 protein stability and reveals a novel therapeutic strategy to enhance the clinical efficacy of immunotherapy.

## Materials and methods

### Analysis of CRISPR Screen

MAGeCK software was used to quantify and test for sgRNA and gene enrichment.^16^^,44^ Trim the sequence reads to remove the constant portion of the sgRNA sequence, and map it to the H1/H2 human genome-wide CRISPR library using the MAGeCK counting module, which calculates read counts for each sgRNA. We used the MAGeCK testing module to identify PD-L1 regulatory factors, and compared to the PD-L1 low group, the PD-L1 high group showed significant enrichment of targeting sgRNA. MAGeCK returned logarithmic changes in sgRNA and genes, representing the enrichment level of the sgRNAs and genes in each cell population.

### Clinical tissue samples and ethics statement

466 cases of paired samples of G.C. tissues and corresponding non-cancerous stomach tissues from Peking University Cancer Hospital were used to detect TRIM28 and PD-L1 expression. The patients included in this study need to meet the following criteria: (1) Histologic identification of the adenocarcinoma. (2) Patients without preoperative chemotherapy, radiotherapy, or immunotherapy. Among them, 466 paired samples were used for the IHC assay, and 98 pairs of samples were used for qRT-PCR detection. We strictly adhere to the ethical guidelines of the Helsinki Declaration. Prior to participation, all patients signed written informed consents and approved by the ethics committee. Details of the clinicopathologic characteristics of these recruited G.C. patients were shown in Table 1.

### Animal experiments

All animals were handled under guidelines approved by the Peking University Cancer Hospital Institutional Animal Care and Use Committee. Male 615 mice (4–6 weeks of age) were purchased from the Tianjin Institute of Hematology. Tumorigenicity assays were performed using subcutaneous mouse models. For the subcutaneous tumor model, mouse-derived MFC gastric cancer cells (1 × 10^5^) were subcutaneously injected into the side abdomen of 615 mice. After tumor-cell inoculation, it is necessary to wait for 1 week before drug intervention. For antibody-based drug intervention, PD-L1/CTLA4 antibody 100 mg (CVP034; Crown Bioscience) or PBS was injected intraperitoneally every 3 days. For drug-based drug intervention, mice were given daily oral doses of Amlexanox 50–100 mg/kg (Selleck Biotech) reconstituted in 1% CMC-Na (Coolaber). It is necessary measure the subcutaneous tumors using a caliper and then calculate the tumor volumes as the formula (length × width^2^)/2.

### Immunohistochemistry (IHC)

IHC assay was performed as described before.^21,45,46^ Briefly, G.C. tissues and corresponding non-cancerous stomach tissues were stained with primary antibodies overnight at 4 °C and followed with the specific HRP-conjugate secondary antibody for 30 min at room temperature. Then, Staining was performed with DAB and counterstained with Mayer’s hematoxylin.

### Co-immunoprecipitation (Co-IP) and immunoblotting

Lyse the whole cells in I.P. buffer which includes NP-40, 50 mM Tris-HCl, pH 7.4, 50 mM EDTA, 150 mM NaCl and protease inhibitor, and centrifuged at 14,000 × *g* at 4 °C for 10 min. Next, collect supernatants and incubate with different antibodies at 4 °C for 2 h, then mix with protein A/G agarose beads overnight at 4 °C on a rotating wheel. After that, wash the beads five times with I.P. buffer at 1000 × *g* for 5 min at 4 °C. Immunoprecipitates were boiled in 2 × SDS loading buffer and then identify the immunoprecipitates by immunoblotting. Immunoblotting assay was performed as described before.^45^ For immunoblotting analysis, lyse the whole cells in RIPA buffer with protease inhibitor and phosphatase inhibitor on ice for 30 min, and then centrifuge the lysates at 12,000 × rpm at 4 °C for 25 min. Measure protein concentrations with BCA kit.

### SUMOylation assay

SUMOylation assay was performed as described before.^21^ Lyse the whole cells in lysis buffer and centrifuge the lysate at 4 °C for 10 min. Next, collect supernatants and incubate with different antibodies at 4 °C for 2 h and then together with protein A/G agarose beads overnight at 4 °C. After that, wash the beads five times with I.P. buffer at 1000 × *g* for 5 min at 4 °C. Immunoprecipitates were boiled in 2 × SDS loading buffer and then analyzed by using western blot assay.

### Statistical analysis

Statistical analyses were conducted using SPSS 22.0 (SPSS, IL, USA) or Prism 8.0 (GraphPad, La Jolla, USA). Survival analysis was conducted using the Kaplan–Meier curves. The Spearman method was used to analyze the correlation between two variables. The Student’s t-test or *Χ*^2^-test was used to study quantitative variables. Data were presented in the form of mean ± S.D. All statistical analyses were two-sided and considered statistically significant as *P*-value < 0.05.

## Supplementary information

Supplemental Data

original data

## Data Availability

The authors declare that the data in this article are available upon reasonable request.
