# Supplementary material for: TRIM28 promotes the escape of gastric cancer cells from immune surveillance by increasing PD-L1 abundance
Source: Signal Transduct Target Ther. 2023 Jun 26;8:246. doi: 10.1038/s41392-023-01450-3 (PMC10290989; doi:10.1038/s41392-023-01450-3)
Supplement: Supplementary file 1 — Supplemental Data [file 41392_2023_1450_MOESM1_ESM.doc]

Supplementary Materials for

**TRIM28 promotes the escape of gastric cancer cells from immune surveillance by increasing PD-L1 abundance**

Xiaoxiao Ma^1,2*^, Shuqin Jia^2*^, Gangjian Wang^1*^, Min Liang^2^, Ting Guo^1^, Hong Du^1^, Sisi Li^2^, Xiaomei Li^1^, Longtao Huangfu^1^, Jianping Guo^3#^, Xiaofang Xing^1#^,

Jiafu Ji^1,2,4#^

Correspondence to: [jijiafu@hsc.pku.edu.cn](mailto:jijiafu@hsc.pku.edu.cn); [xingxiaofang@bjmu.edu.cn](mailto:xingxiaofang@bjmu.edu.cn);

[guojp6@mail.sysu.edu.cn](mailto:guojp6@mail.sysu.edu.cn)

**This PDF file includes:**

Materials and Methods

Reference

Figures. S1 to S9

**Materials and methods**

**CRISPR Screens for regulators of PD-L1**

We performed CRISPR screens to identify regulators of PD-L1. We first transduced 1 × 10^8^ N87 cells with our H1/H2 human genome-wide CRISPR library at multiplicity of infection of 0.3 to ensure that most transduced cells received only 1 virion. We cultured the transduced cells for 2 days in DMEM full media, selected with 2 μg/mL puromycin for 2 days, and cultured for an additional 2 days to allow depletion of residual protein of the targeted genes. The resulting cells were dissociated by TrypLE treatment and incubated with APC conjugated anti-PDL1(BD biosciences, clone MIH5) or APC conjugated IgG2a as control for 1 hour on ice. Then the cells were washed and re-suspended in ice-cold PBS buffer. We then flow-sorted 2× 10^7^ cells for PDL1^pos^ and PD-L1^neg^ population, respectively, which were collected and pelleted for genomic DNA extraction. Genomic DNA extraction and gRNA library construction were performed as described before. Briefly, we extracted genomic DNA from each sorted population by phenol–chloroform extraction, and then performed two rounds of PCR to construct the barcode-indexed sequencing library for each sample. We sequenced each library at an approximately 300× average coverage over the CRISPR library.

**Cell lines and Cell culture**

The G.C. cell lines N87, SGC-7901, MGC-803, AGS, BGC-823 were obtained from the Cell Research Institute (Shanghai, China). The cells were cultured in high-glucose Dulbecco’s modified eagle media (DMEM,Gibco, Grand Island, NY, USA) replenished with 10% (v/v) fetal calf serum (Gibco) and incubated in a humidified atmosphere containing 5% CO_2_ at 37℃.

**Antibodies, reagents and plasmids**

The primary antibodies used in immunohistochemistry, western blot and confocal assays included antibodies against TRIM28 (SAB2701980, SIGMA), PD-L1 (66248-1-Ig, Proteintech, #13684, Cell Signaling Technology) and GAPDH (TA-08, ZSGB-BIO). The antibodies used in western blot analysis including antibodies against CD4(#75508), CD8(#98941), p-TBK1(#5483), TBK1(#3013), IRF1(#8478), Myc(#2276), Flag(#14793), HA(#3724), His(#12698), p-mTOR(#2971), p-S6K1(#9234), p-4E-BP1(#2855), LaminB1(#12586) were all bought from Cell Signaling Technology (Cell Signaling Technology, Beverly, USA). MG132(S2619), 2-D08 (S8696) and Amlexanox(S3648) were purchased from Selleck Chemicals. CHX(A8244) was purchased from APExBIO Technology. TRIM28 expression plasmid was purchased from Genecopoia Technology. HA-SUMO1, HA-SUMO2, HA-SUMO3, and His-UBC9 were provided by Dr Lihui Han (Shandong University).

**Quantitative real-time PCR**

Total RNA was extracted from G.C. tissues or G.C. cells using Trizol Reagent according to the manufacturer's instructions and then was reverse transcribed into cDNA. Quantitative real-time PCR was performed using SYBR Green PCR Master Mix with different primers and reactions were performed on ABI 7500 System (Applied Biosystems, Foster City, CA, USA). Primers for human TRIM28 gene were forward: 5′-TTTCATGCGTGATAGTGGCAG-3′, reverse: 5′-GCCTCTACAC

AGGTCTCACAC-3′. Primers for human PD-L1 gene were forward: 5′-TCAATGCCCCATACAACAAA-3′, reverse: 5′-TGCTTGTCCAGATGACTTCG

-3′. Relative gene expression levels were normalized to GAPDH. Primers for GAPDH gene were forward: 5′-AAGGTGAAGGTCGGAGTCAA-3′, reverse: 5′-AATGAAG

GGGTCATTGATGG-3′. The relative mRNA levels of target genes were obtained by using the 2^–ΔΔCt^ method with all assays performed in triplicate.

**Immunofluorescence and Confocal**

G.C. cells were fixed in 4% paraformaldehyde for 15 minutes and permeabilized with 0.5% Triton-X 100 for 10 minutes, and then kept in blocking buffer for 1 h. Then, the cells were incubated with primary antibodies overnight at 4℃, followed by stained with secondary antibodies. Nuclei were stained with DAPI. After that, the cells were visualized using laser scanning confocal microscope LSM 780 with Zen software (Carl Zeiss, Toronto, ON, Canada).

**In vitro binding assay**

According to the manufacturer's protocol, the TNT Quick Coupled Transcription and Translation System kit (Promega, Madison, WI, USA) was used to detect the direct interaction between TRIM28 and PD-L1.

**Subcellular fractionation**

According to the manufacturer's protocol, the Nuclear and Cytoplasmic Protein Extraction Kit (Beyotime, Jiangsu, China) was used to extract and isolate nuclear and cytoplasmic proteins from G.C. cells, and subjected to western blot assay.

**T cell–mediated tumor cell killing assays**

T cell–mediated tumor cell killing assays were performed as previously described^1^. Primary human T cells isolated from healthy human peripheral blood were activated with a CD3/CD28 antibody (100 ng/mL) and interleukin-2 (10 ng/mL). The tumor cells were seeded into precoated 96-well plate at a density of 2,000 cells per well. Then the activated T cells were co-cultured with tumor cells at a ratio of 10:1. After that, cells were washed with PBS and detected by a CCK-8 Kit at 0, 24, 48, and 72 h according to the manufacturer’s instructions. Besides, the tumor cells and the activated T cells were co-cultured in 6-well plate at 10:1 ratio for several days. After that, cells were washed with PBS for three times to remove T cells, followed by staining with crystal violet. Finally, scan and quantify the dried plates.

**RNA-sequencing**

RNA-seq was carried out in SGC-7901 cells with overexpression of TRIM28. GO enrichment analysis was used to analyze the biological significance, including biological processes, cellular components and molecular functions. KEGG enrichment analysis was applied to analyze the pathway and identify the critical pathways. P<0.05 was considered statistically significant.

**Sources of public database**

We downloaded the information of G.C. tissues and corresponding non-cancerous stomach tissues from the GDC TCGA data portal (<https://portal.gdc.cancer.gov/>) and GSE66229^2^ dataset from the GEO database (https://www.ncbi.nlm.nih.gov/geo/). Then, we calculated the expression level of TRIM28 and PD-L1 and conducted the difference significance test between the tumor group and the normal group.

Overall, 27 TCGA solid tumors (carcinomas) ACC, BLCA, BRCA, CESC, CHOL, COAD, READ, ESCA, GBM, STAD, HNSC, KICH, KIRC, KIRP, LGG, LIHC, LUAD, LUSC, MESO, OV, PAAD, PCPG, PRAD, SARC, SKCM, THCA, UCEC, were used in the study. Clinical and genome data were downloaded from the GDC TCGA data portal. Transcriptomic data were downloaded from the USCS XENA portal (<https://xena.ucsc.edu/>), then the values were transformed into transcripts per kilobase million (TPM) values., we obtained the GSE84437^3^ dataset from the GEO database and the 45 cases of gastric cancer treated with immunotherapy from the SRA database (https://www.ncbi.nlm.nih.gov/sra/) (PRJEB25780)^4^.

In addition to the dataset of GCs treated with anti-PD1 antibody, 9 cohorts receiving different types of immunotherapies were included in our study. The 9 cohorts were (1) IMvigor210 (advanced urothelial cancer with anti-PD-L1 antibody) ^5^, (2) GSE78220 (metastatic melanoma with anti-PD1 antibody) ^6^, (3) GSE115821 (melanoma with anti-PD1or CTLA4 antibody^7^, (4) GSE100797 (Melanoma receiving adoptive T cell therapy)^8^, (5) GSE173839 (BRCA with PD-L1 antibody)^9^, (6) GSE111636 (COAD with PD1 antibody), (7) GSE67501 (KIRC with PD-L1 antibody)^10^, (8) Allen et al (Metastatic Melanoma with CTLA4 antibody)^11^, (9) and Pender cohort (pan-cancer treated with various types of immunotherapies)^12^.

**Immune cell infiltration analysis based on bulk RNA sequencing data**

ESTIMATE algorithm was used to evaluate the relationship between TRIM28 expression and immune infiltration^13^. TIMER 2.0 (http://timer.cistrome.org/) is a powerful platform that integrates a variety of algorithms for calculating infiltration of a variety of different immune cells^14^. In the present study, we investigated the association between TRIM28 expression and tumor-immune infiltrations, including CD8^+^ T cells, myeloid-derived suppressor cells (MDSCs) based on TIDE^15^ using the ‘Gene Module’.

**Reference**

1. Li H, et al. MET Inhibitors Promote Liver Tumor Evasion of the Immune Response by Stabilizing PDL1. *Gastroenterology*. **156**(6)**:** 1849-1861 e1813 (2019).

2. Oh SC, et al. Clinical and genomic landscape of gastric cancer with a mesenchymal phenotype. *Nat Commun.* **9**(1)**:** 1777 (2018,).

3. Yoon SJ, et al. Deconvolution of diffuse gastric cancer and the suppression of CD34 on the BALB/c nude mice model. *BMC cancer*. **20**(1)**:** 314 (2020).

4. Kim ST, et al. Comprehensive molecular characterization of clinical responses to PD-1 inhibition in metastatic gastric cancer. *Nat Med.* **24**(9)**:** 1449-1458 (2018).

5. Mariathasan S, et al. TGFβ attenuates tumour response to PD-L1 blockade by contributing to exclusion of T cells. *Nature*. **554**(7693)**:** 544-548 (2018).

6. Hugo W, et al. Genomic and Transcriptomic Features of Response to Anti-PD-1 Therapy in Metastatic Melanoma. *Cell*. **165**(1)**:** 35-44 (2016).

7. Auslander N, et al. Robust prediction of response to immune checkpoint blockade therapy in metastatic melanoma. *Nat Med.* **24**(10)**:** 1545-1549 (2018).

8. Lauss M, et al. Mutational and putative neoantigen load predict clinical benefit of adoptive T cell therapy in melanoma. *Nat Commun.* **8**(1)**:** 1738 (2017).

9. Pusztai L, et al. Durvalumab with olaparib and paclitaxel for high-risk HER2-negative stage II/III breast cancer: Results from the adaptively randomized I-SPY2 trial. *Cancer cell*. **39**(7)**:** 989-998.e985 (2021).

10. Ascierto ML, et al. The Intratumoral Balance between Metabolic and Immunologic Gene Expression Is Associated with Anti-PD-1 Response in Patients with Renal Cell Carcinoma. *Cancer Immunol Res.* **4**(9)**:** 726-733 (2016).

11. Van Allen EM, et al. Genomic correlates of response to CTLA-4 blockade in metastatic melanoma. *Science (New York, NY)*. **350**(6257)**:** 207-211 (2015).

12. Pender A, et al. Genome and Transcriptome Biomarkers of Response to Immune Checkpoint Inhibitors in Advanced Solid Tumors. *Clin Cancer Res.* **27**(1)**:** 202-212 (2021).

13. Yoshihara K, et al. Inferring tumour purity and stromal and immune cell admixture from expression data. *Nat Commun.* **4:** 2612 (2013).

14. Li T, et al. TIMER2.0 for analysis of tumor-infiltrating immune cells. *Nucleic Acids Res.* **48**(W1)**:** W509-w514 (2020).

15. Jiang P, et al. Signatures of T cell dysfunction and exclusion predict cancer immunotherapy response. *Nat Med.* **24**(10)**:** 1550-1558 (2018).

**Figure. S1.**


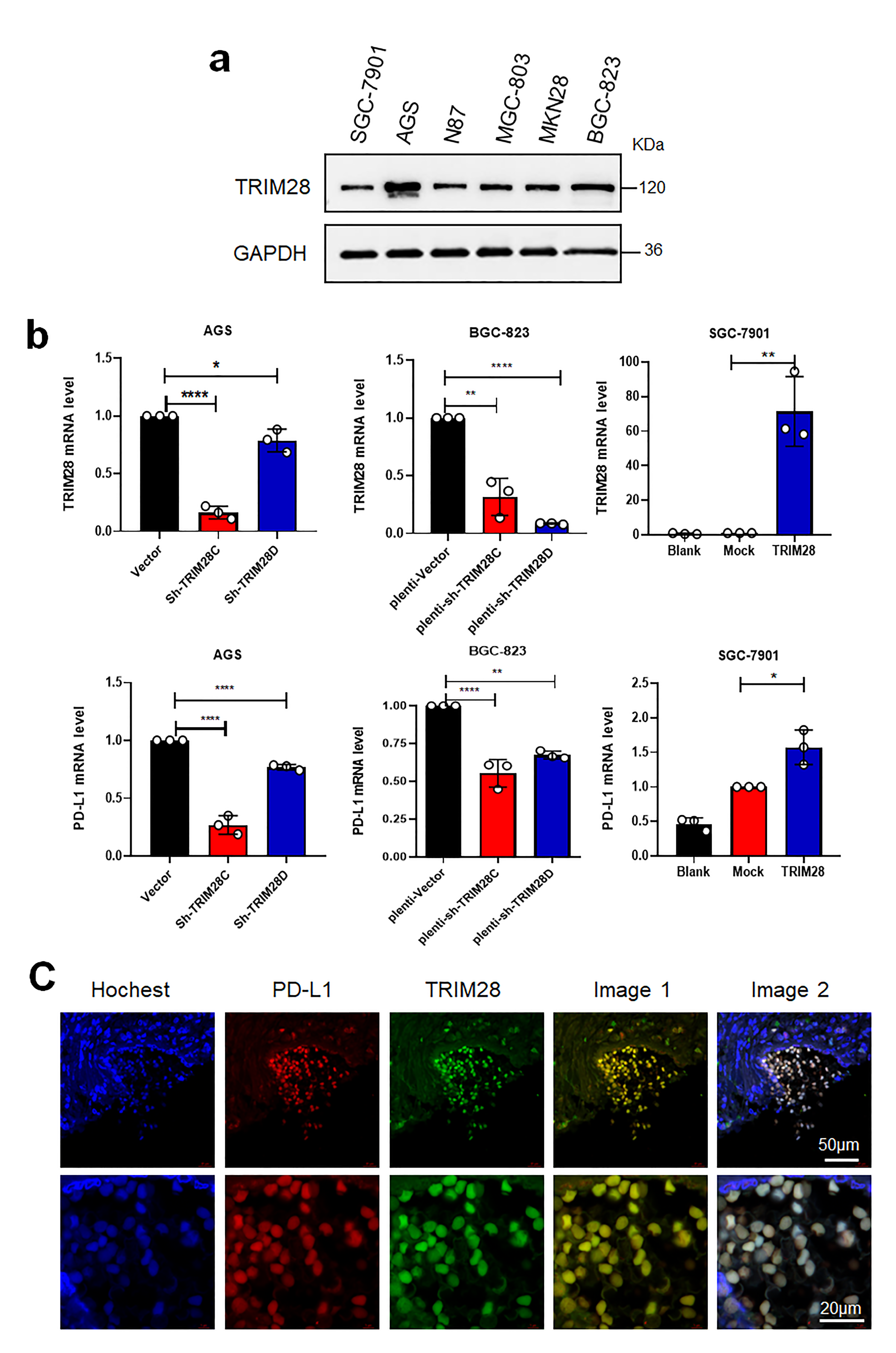


**Fig. S1. TRIM28 regulates PD-L1 in G.C. cells. a,** Western blot analysis of the basic protein levels of TRIM28 in multiple G.C. cells. **b,** mRNA levels of TRIM28 and PD-L1 were detected by qRT-PCR and further quantitatively analyzed. **c**, Confocal microscopy analysis showing colocalization of PD-L1 (Red) and TRIM28 (Green) in tissue microarray. Bars = means ± SD; n = 3; ns, no significance; *P < 0.05; **P < 0.01; ***P < 0.001; ****P < 0.0001.

**Figure. S2.**


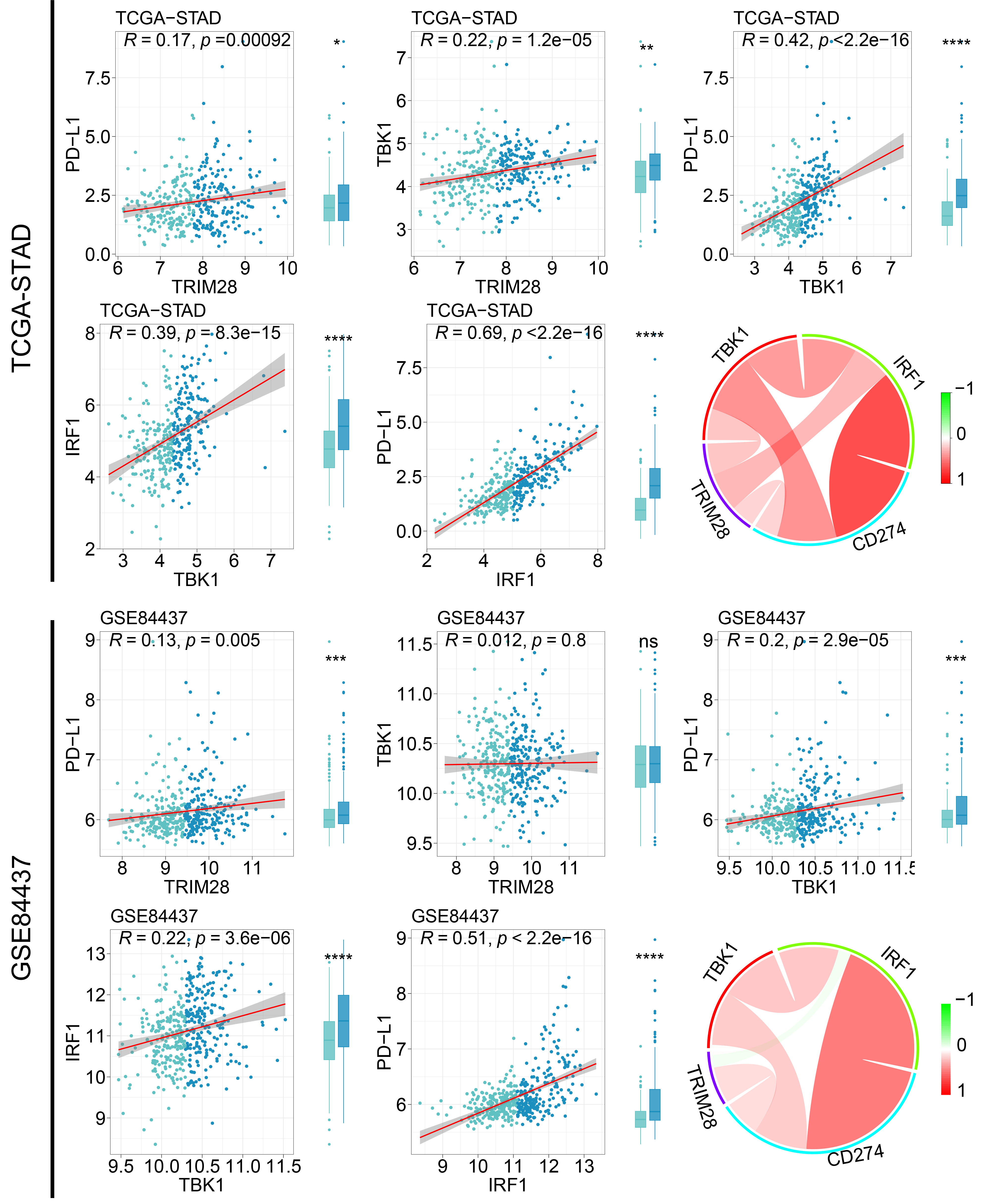


**Fig. S2.** Correlation analysis of TRIM28 and PD-L1, TBK1, IRF1 from TCGA and GEO datasets.

**Figure. S3.**


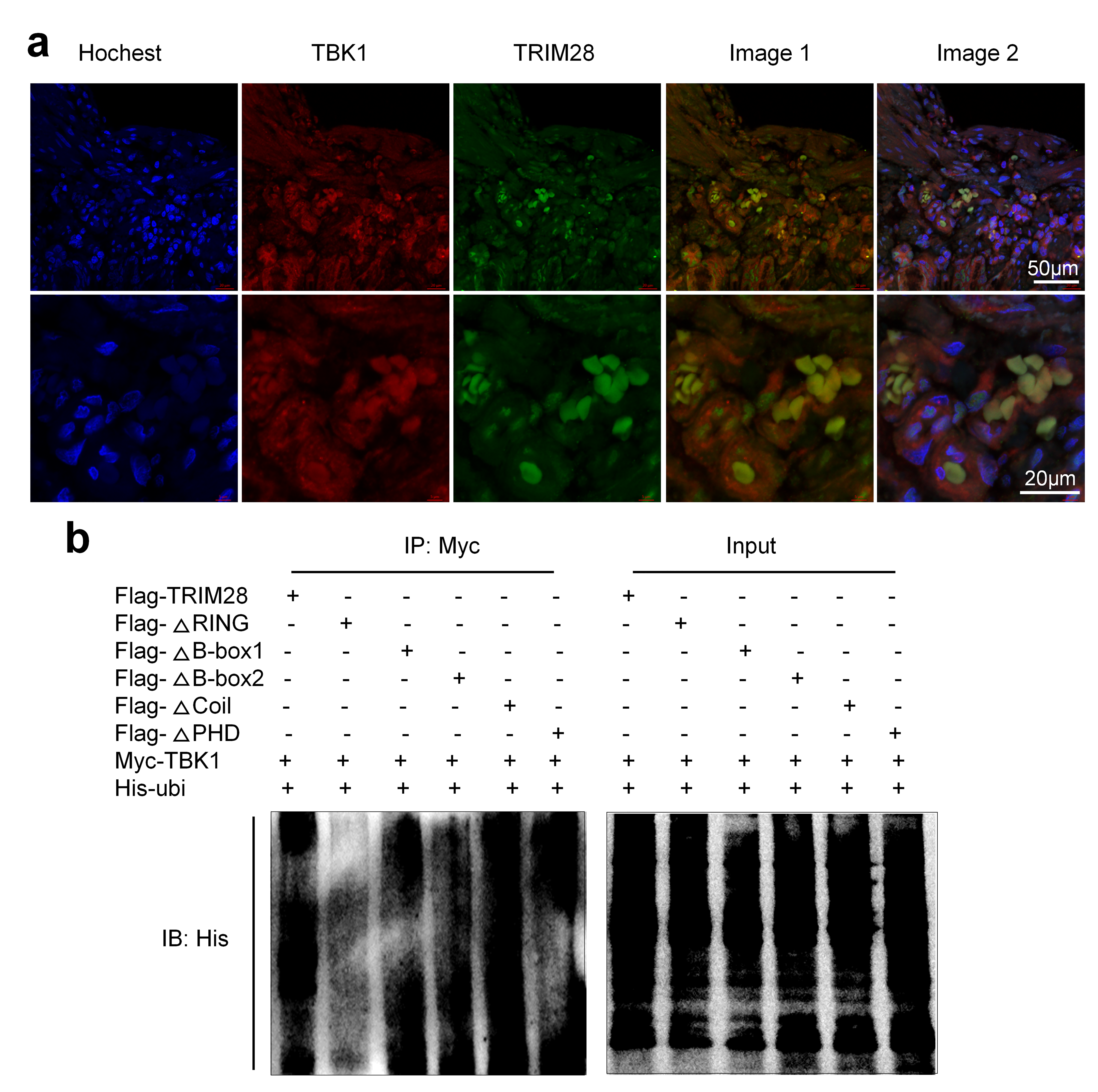


**Fig. S3. TRIM28 targets TBK1. a**, Confocal microscopy analysis showing colocalization of TBK1 (Red) and TRIM28 (Green) in tissue microarray. **b,** Co-IP analysis of the ubiquitination of TBK1 in SGC-7901 cells which were co-transfected with Myc-TBK1 plasmid, His-ubi plasmid, Flag-TRIM28 full-length or truncation mutant plasmid.

**Figure. S4.**

**
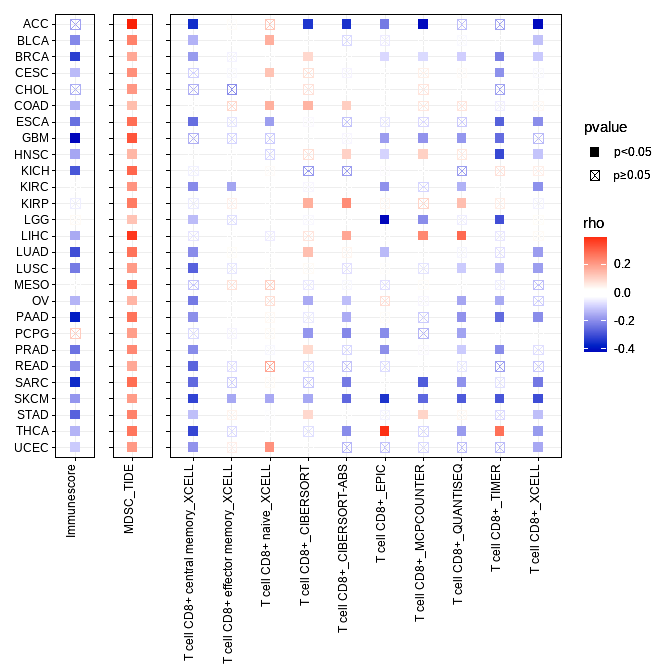
**

**Fig. S4.** The correlations of TRIM28 with CD8^+^ T cell infiltration, MDSC based on TIDE and immunescore based ESTIMATED algorithm in various cancers.

**Figure. S5.**


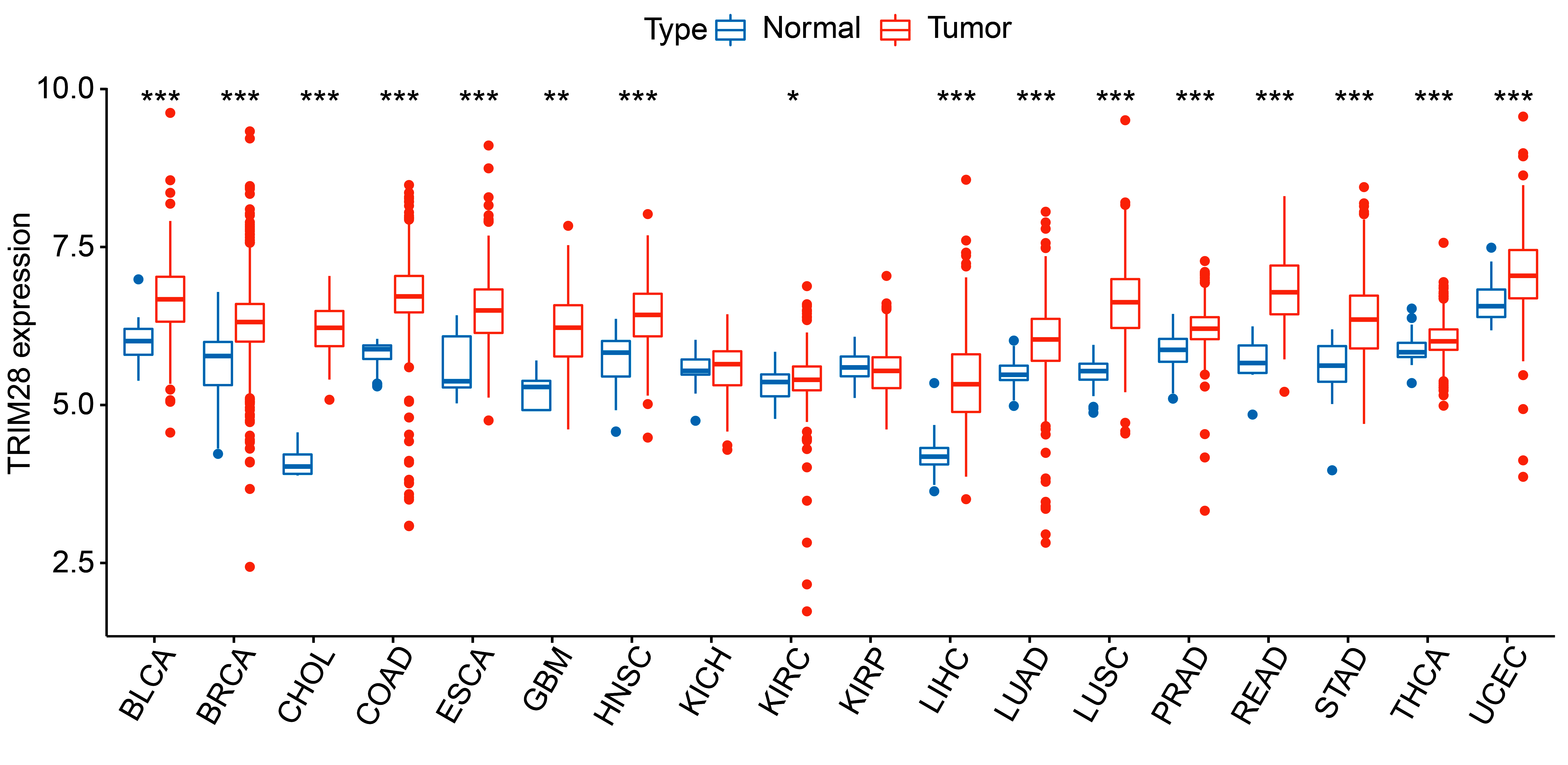


**Fig. S5.** Analysis of TRIM28 expression between the tumor group and adjacent normal group in 18 cancer types from TCGA.

**Figure. S6.**


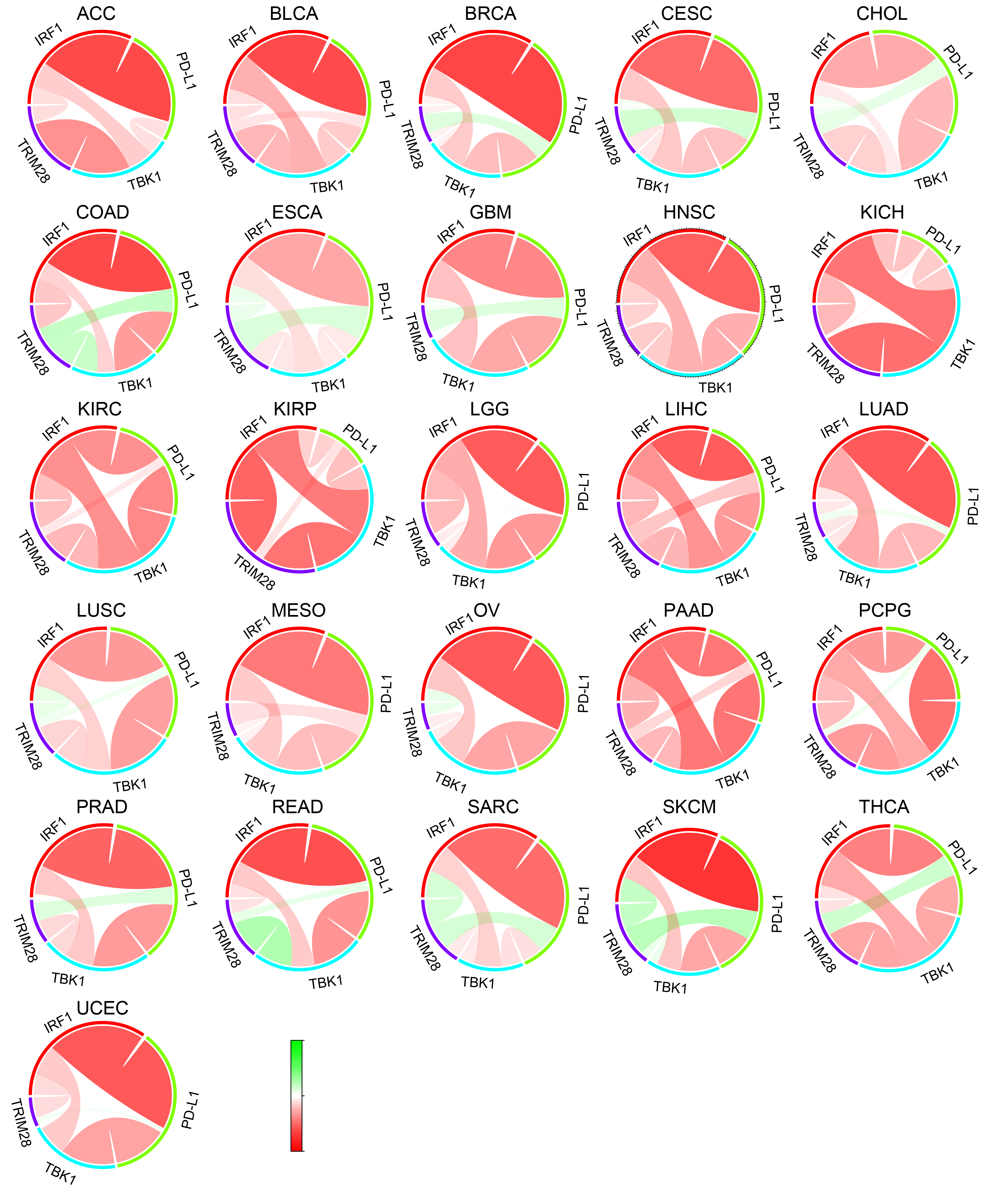


**Fig. S6.** The correlation of TRIM28 with the expression levels of TBK1, IRF1 and PD-L1 in TCGA multiple cancer types.

**Figure. S7.**


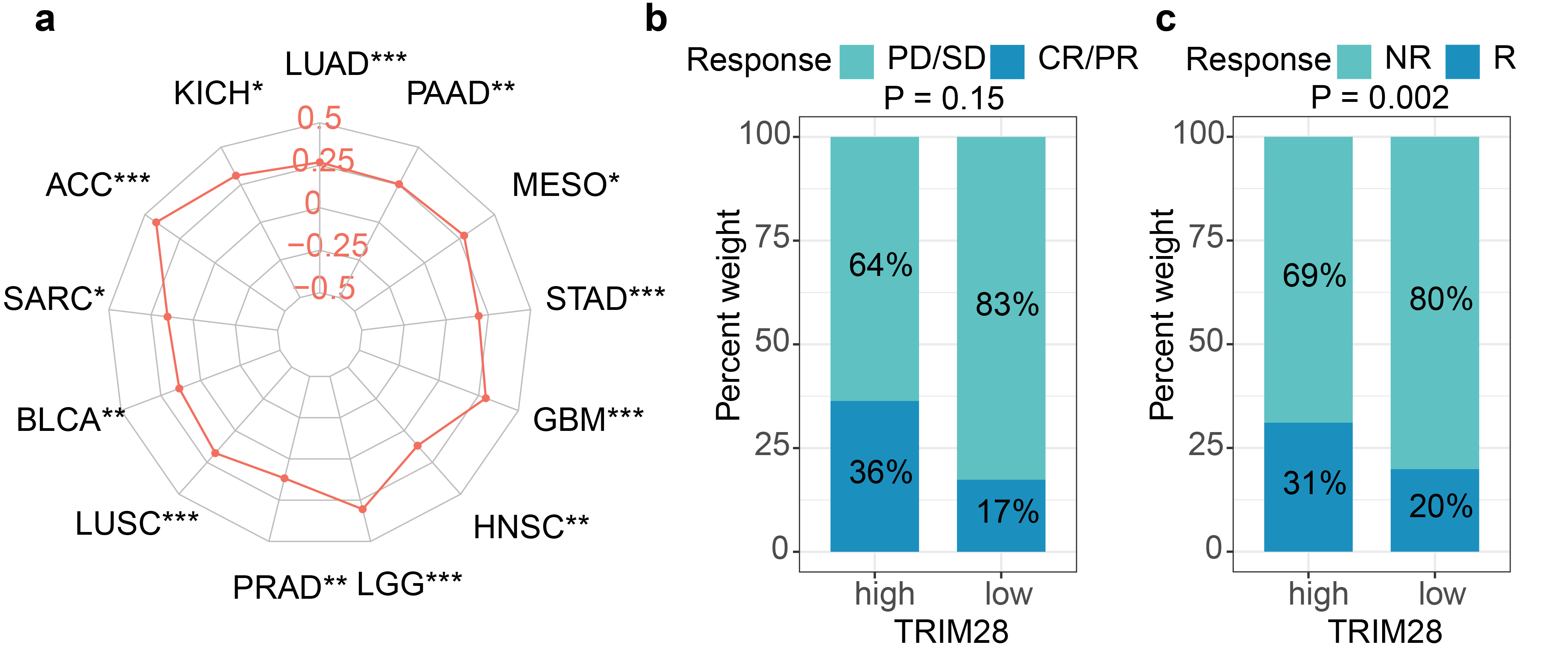


**Fig. S7. The data of pan-cancer analysis. a**, The correlation of TRIM28 with TMB in 13 cancer types. **b**, Analysis of the immunotherapy efficiency of G.C. patients with high levels of TRIM28 or with low levels of TRIM28 from PRJEB25780 dataset. **c,** Analysis of the immunotherapy efficiency of pan-cancer patients from 9 cohorts receiving different types of immunotherapies with high levels of TRIM28 or with low levels of TRIM28.

**Figure. S8.**


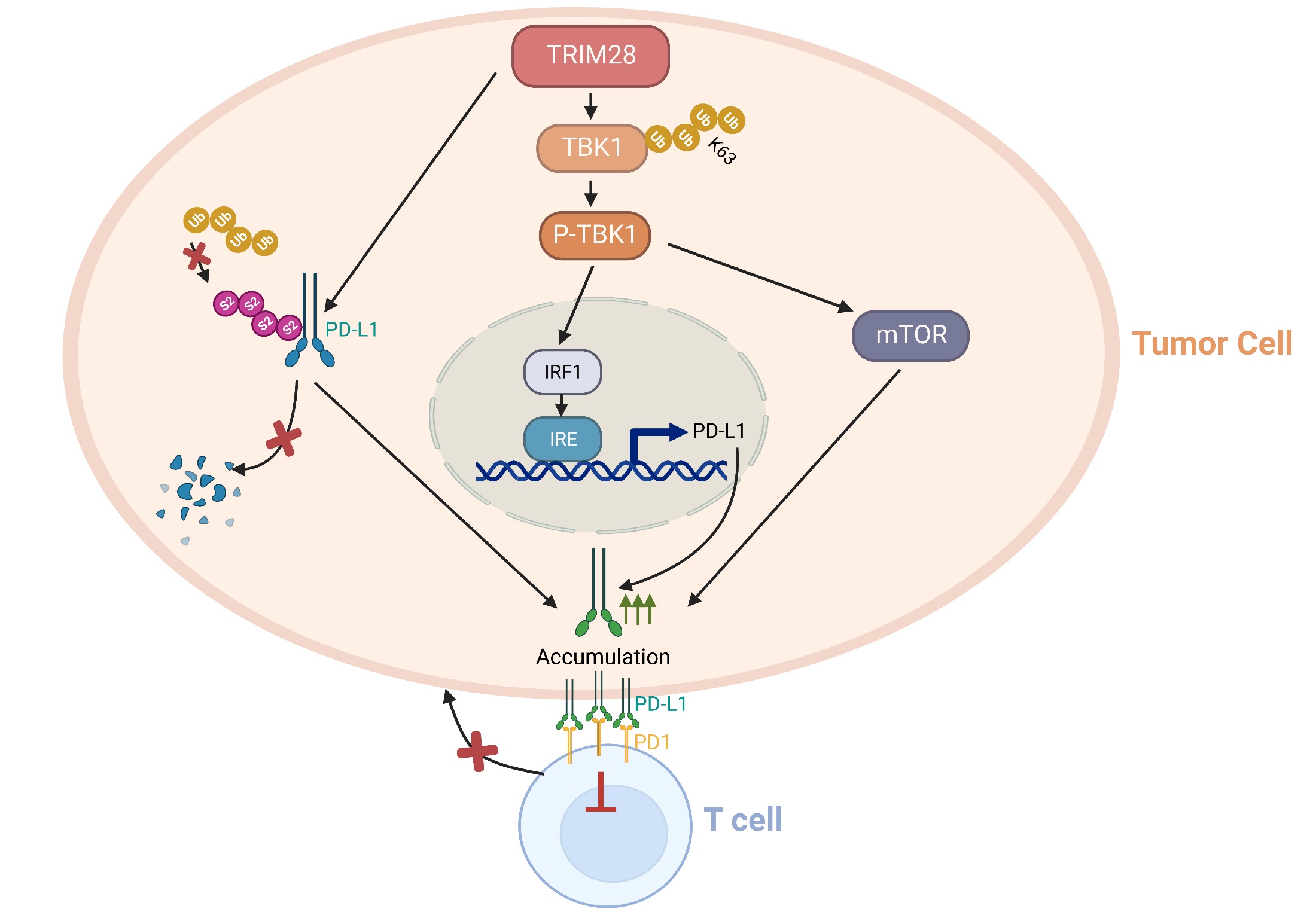


**Fig. S8.** Working model illustrating the role of TRIM28 in regulating PD-L1. TRIM28 directly interacts with PD-L1, promotes SUMOylation of PD-L1, and inhibits PD-L1 ubiquitination; thus, preventing the degradation and increasing the cellular abundance of PD-L1. TRIM28 also interacts with and activates TBK1 by enhancing its K63-linked polyubiquitination, leading to the activation of TBK1-IRF1 or/and TBK1-mTOR pathways that ultimately results in increased PD-L1 expression in G.C. Figure was created using BioRender.

**Figure. S9.**


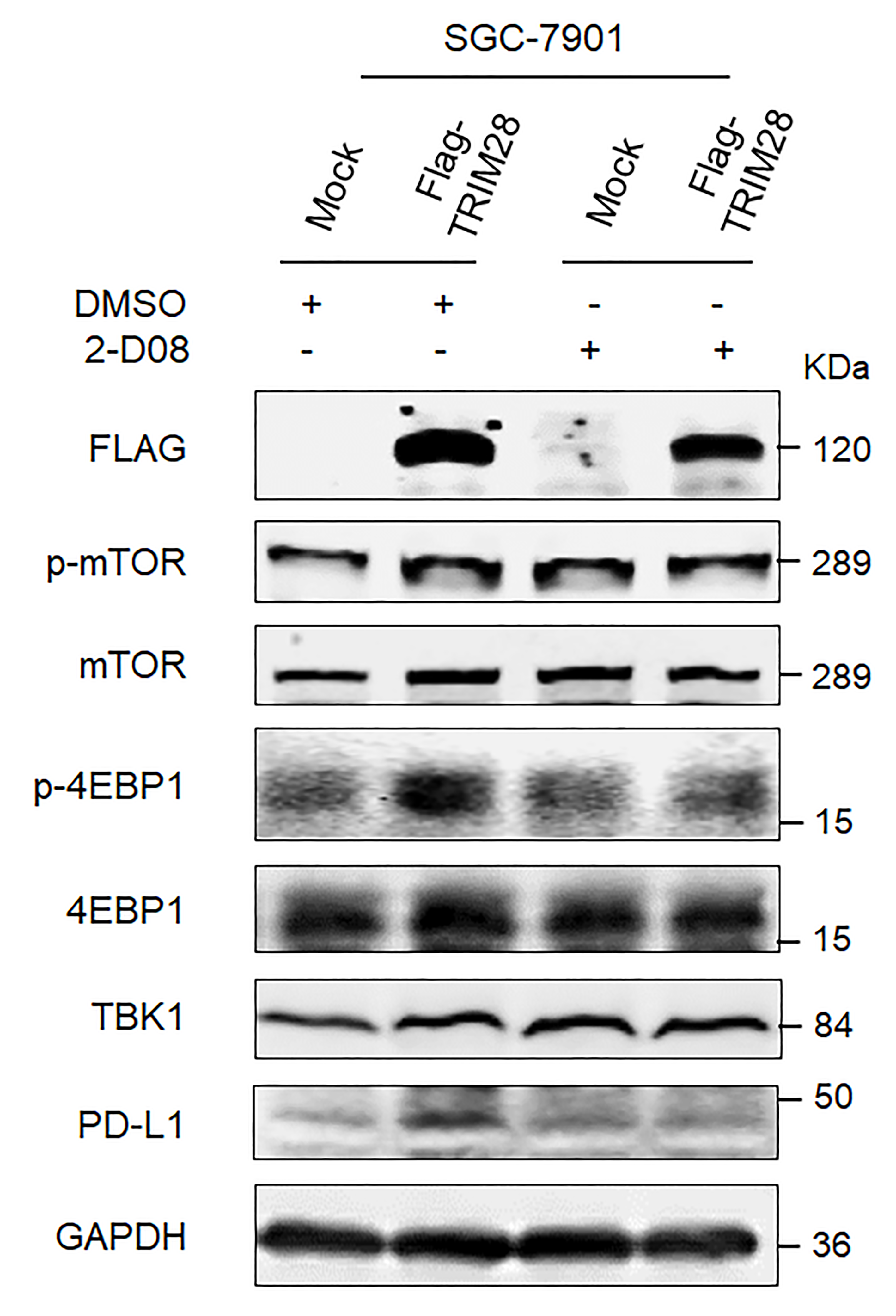


**Fig. S9.** The effect of SUMO inhibitor on TRIM28 mediated pathways and PD-L1 expression in SGC-7901.
