# Supplementary material for: TRIM28 promotes the escape of gastric cancer cells from immune surveillance by increasing PD-L1 abundance
Source: Signal Transduct Target Ther. 2023 Jun 26;8:246. doi: 10.1038/s41392-023-01450-3 (PMC10290989; doi:10.1038/s41392-023-01450-3)
Supplement: Supplementary file 2 [file 41392_2023_1450_MOESM2_ESM.docx]

Fig. 1

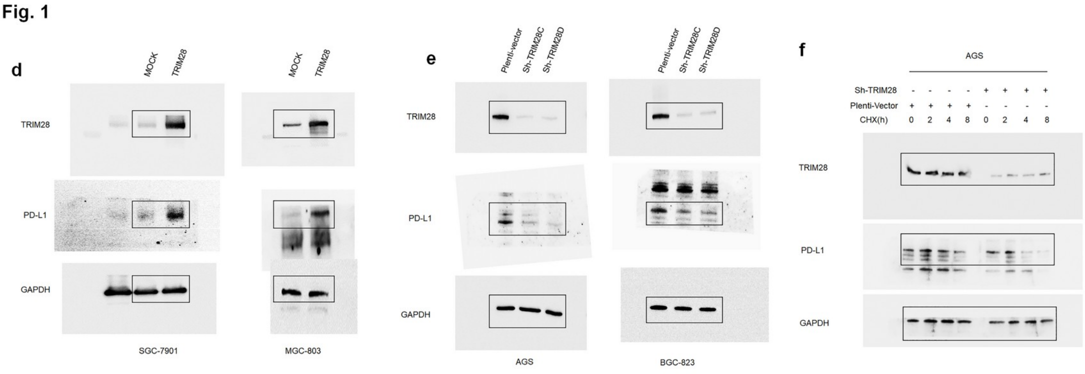

d
MOCK
TRIM28
TRIM28
PD-L1
GAPDH
SGC-7901
MGC-803
e
Plenti-vector
Sh-TRIM28C
Sh-TRIM28D
TRIM28
PD-L1
GAPDH
AGS
BGC-823
f
AGS
Sh-TRIM28
Plenti-Vector
CHX(h)
0 2 4 8 0 2 4 8
TRIM28
PD-L1
GAPDH

Fig. 2

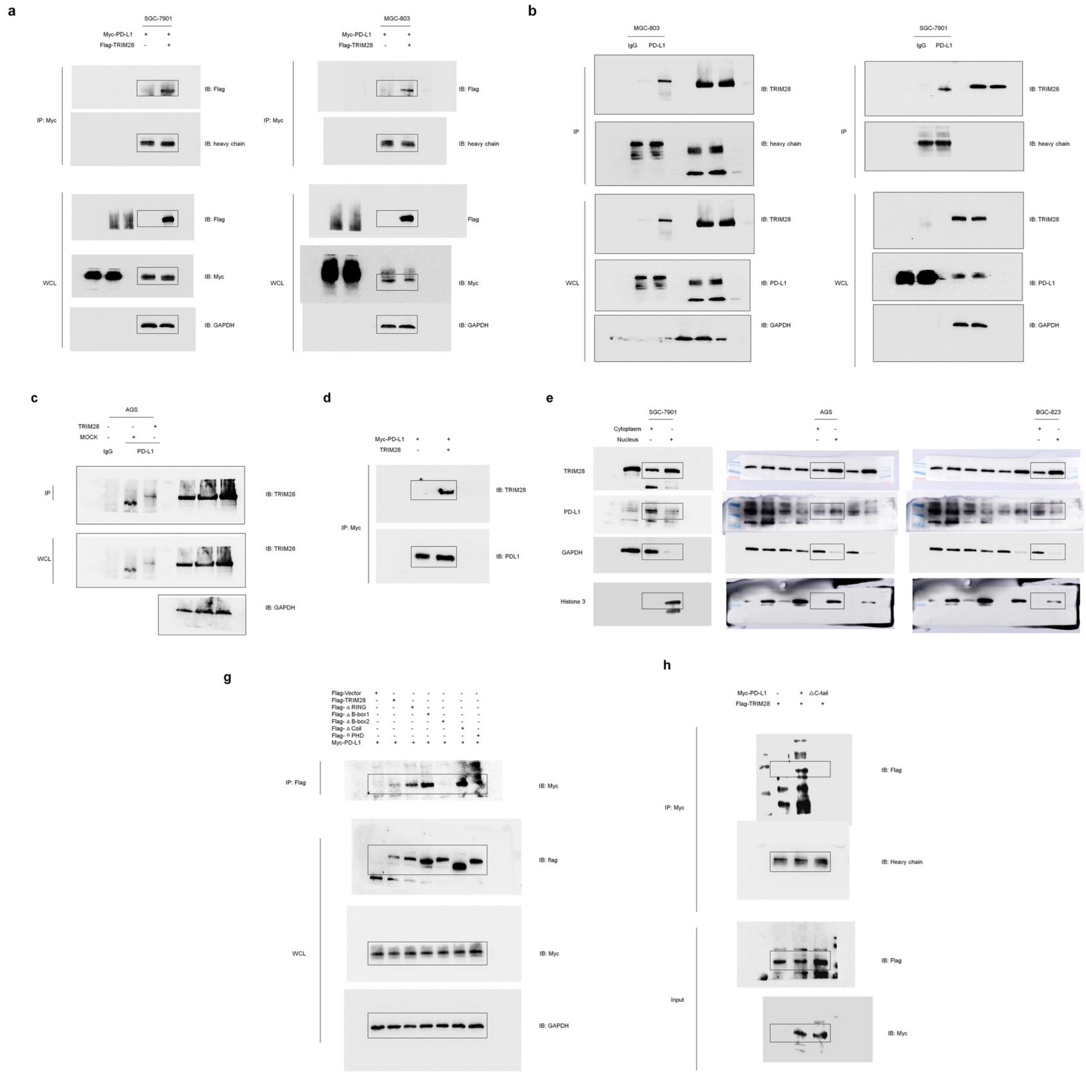

a
SGC-7901
MGC-803
Myc-PD-L1
Flag-TRIM28
IP: Myc
IB: Flag
IB: heavy chain
WCL
IB: Myc
IB: GAPDH
b
IgG
PD-L1
IP
IB: TRIM28
IB: PD-L1
c
AGS
TRIM28
MOCK
IgG
PD-L1
WCL
d
IB: PDL1
e
Cytoplasm
Nucleus
AGS
BGC-823
Histone 3
g
Flag-Vector
Flag-TRIM28
Myc-PD-L1
IP: Flag
IB: flag
h
△C-tail
IB: Heavy chain
Input

Fig. 3

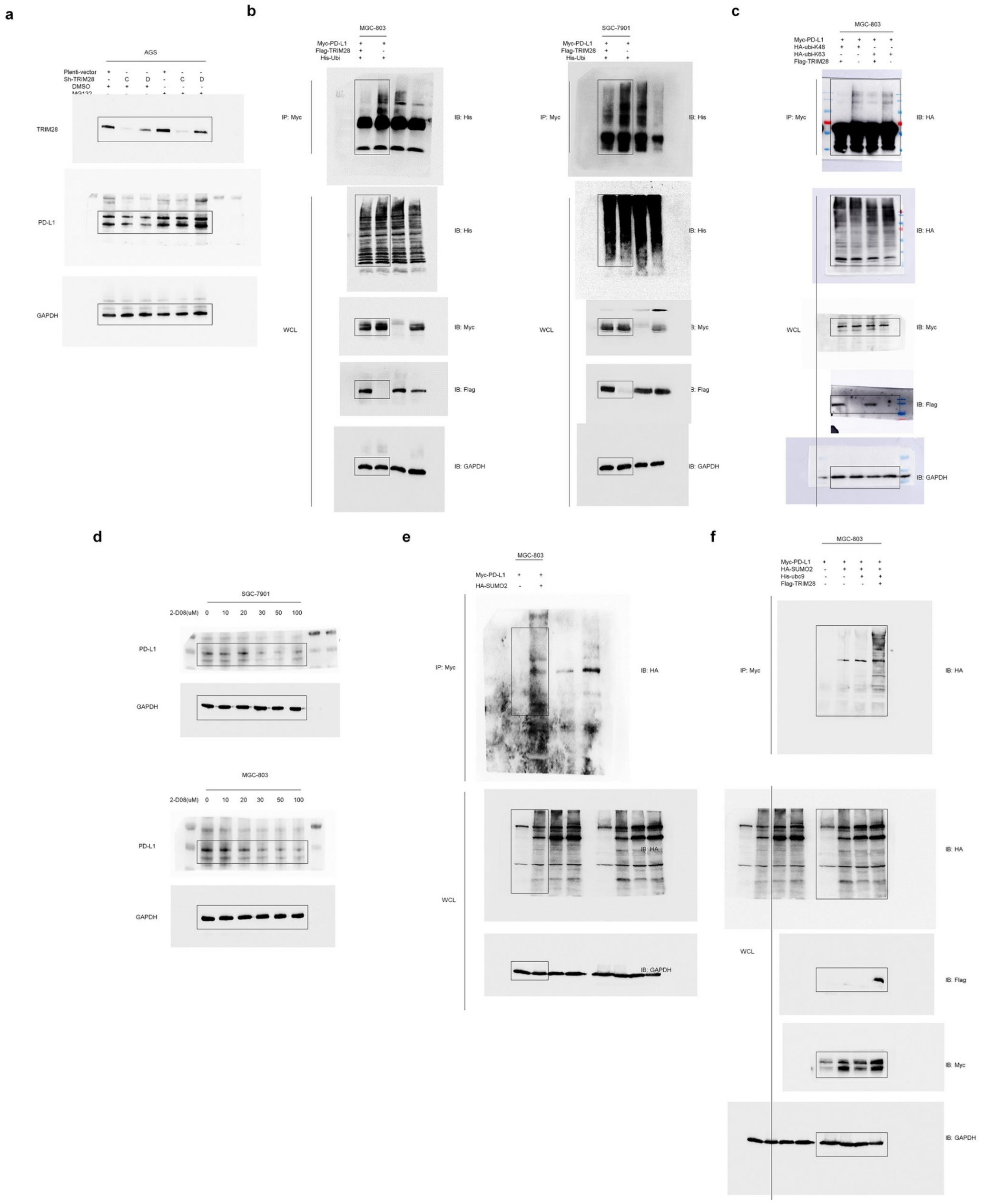
a
AGS
Plenti-vector
Sh-TRIM28
DMSO
TRIM28
PD-L1
GAPDH
b
MGC-803
Myc-PD-L1
Flag-TRIM28
His-Ubi
IP: Myc
IB: His
WCL
IB: Myc
IB: Flag
IB: GAPDH
SGC-7901
c
MGC-803
Myc-PD-L1
HA-ubi-K48
HA-ubi-K63
Flag-TRIM28
IB: HA
d
SGC-7901
2-D08(uM) 0 10 20 30 50 100
MGC-803
e
HA-SUMO2
f
His-ubc9

Fig. 4

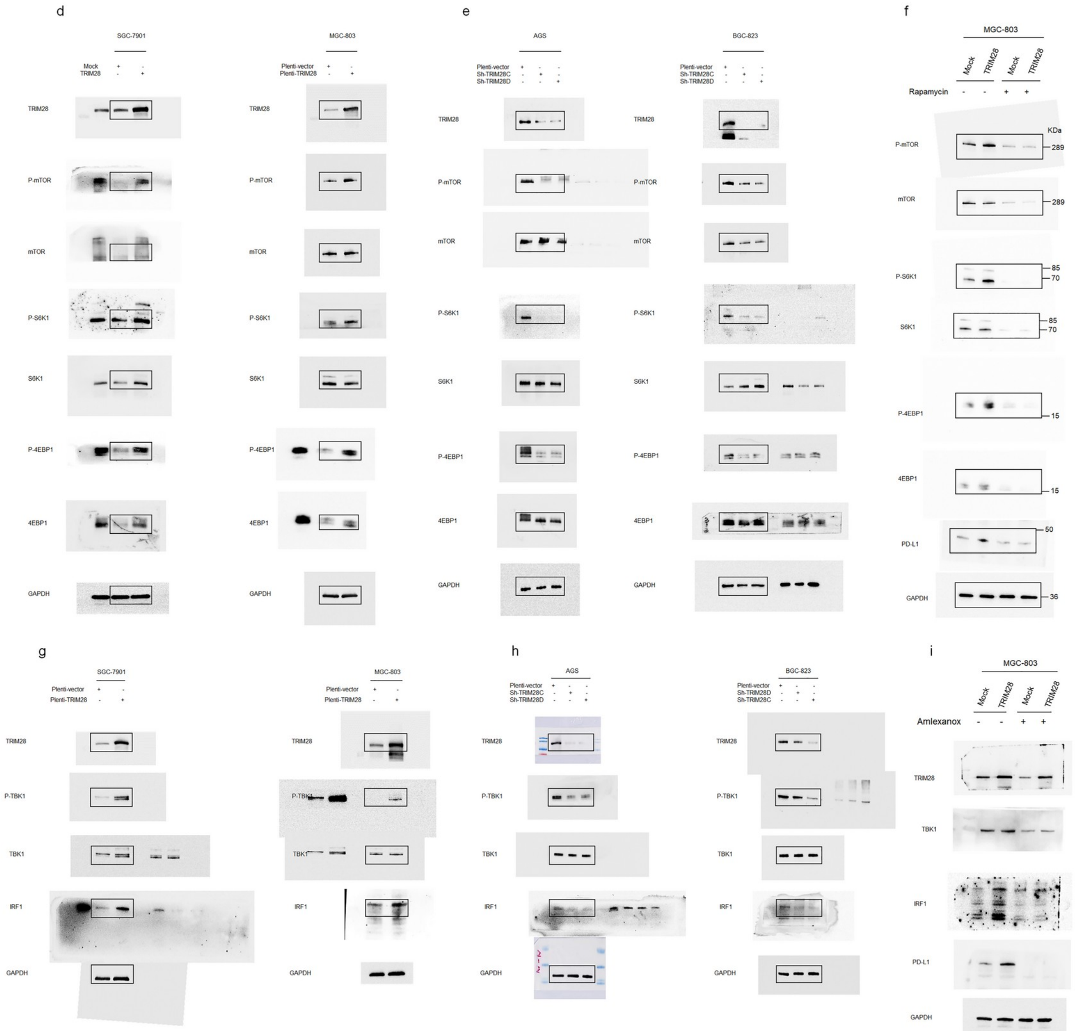
d
SGC-7901
Mock
TRIM28
MGC-803
Plenti-vector
Plenti-TRIM28
TRIM28
P-mTOR
mTOR
P-S6K1
S6K1
P-4EBP1
4EBP1
GAPDH
e
AGS
BGC-823
Plenti-vector
Sh-TRIM28C
Sh-TRIM28D
f
MGC-803
Mock
TRIM28
Rapamycin
KDa
289
85
70
15
50
36
PD-L1
g
SGC-7901
MGC-803
P-TBK1
TBK1
IRF1
h
AGS
BGC-823
i
MGC-803
Amlexanox

Fig. 5

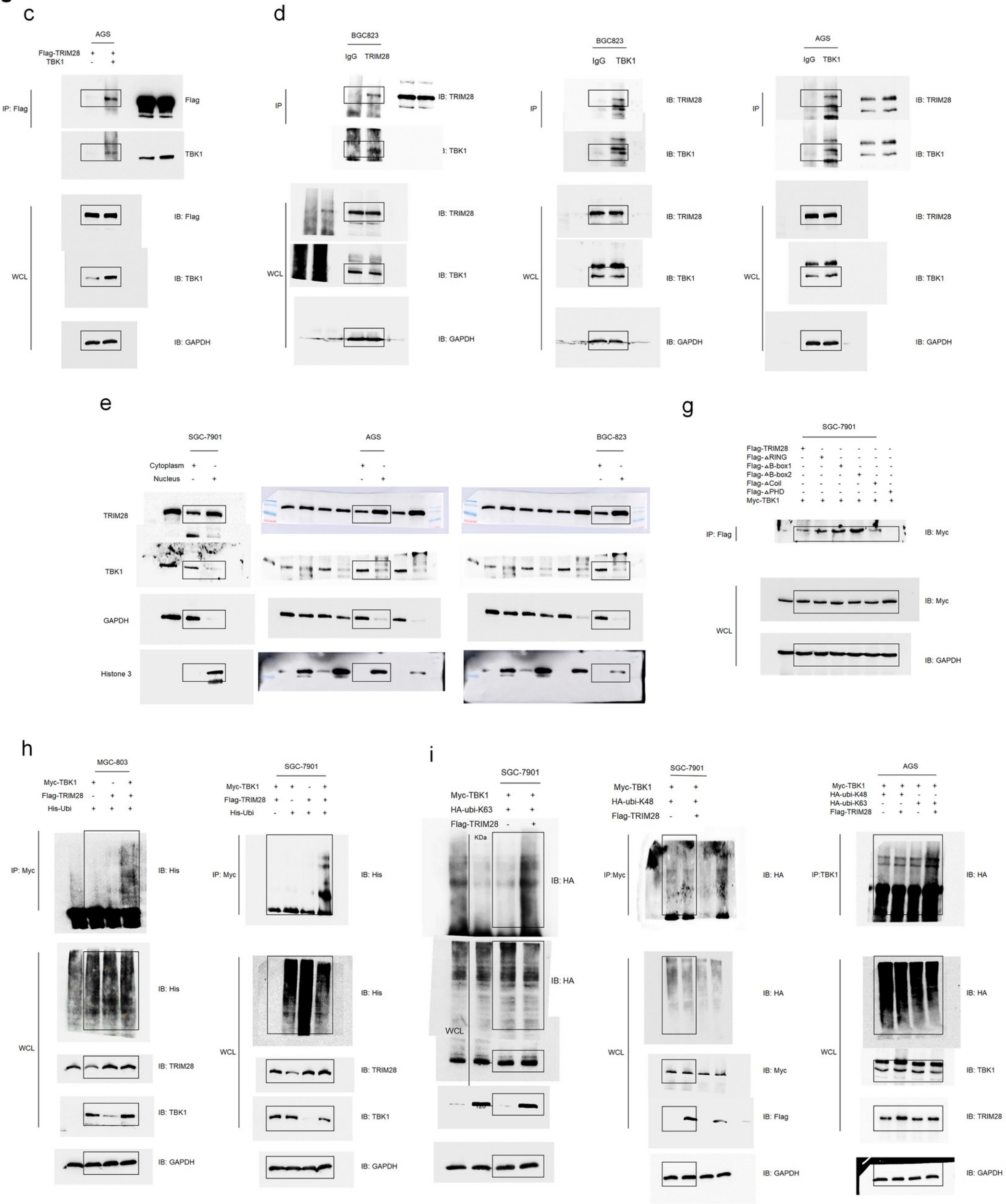
c
AGS
Flag-TRIM28
TBK1
IP: Flag
Flag
TBK1
IB: Flag
WCL
IB: TBK1
IB: GAPDH
d
BGC823
IgG TRIM28
IP
IB: TRIM28
IB: TBK1
WCL
IB: TRIM28
IB: TBK1
IB: GAPDH
BGC823
IgG TBK1
AGS
IgG TBK1
e
SGC-7901
Cytoplasm
Nucleus
AGS
BGC-823
TRIM28
TBK1
GAPDH
Histone 3
g
SGC-7901
Flag-TRIM28
Flag-ΔRING
Flag-ΔB-box1
Flag-ΔB-box2
Flag-ΔCoil
Flag-ΔPHD
Myc-TBK1
IP: Flag
IB: Myc
WCL
IB: GAPDH
h
MGC-803
Myc-TBK1
Flag-TRIM28
His-Ubi
IP: Myc
IB: His
WCL
IB: TRIM28
IB: TBK1
IB: GAPDH
SGC-7901
i
SGC-7901
Myc-TBK1
HA-ubi-K63
Flag-TRIM28
KDa
IB: HA
WCL
SGC-7901
HA-ubi-K48
IP:Myc
IB: Myc
IB: Flag
AGS
HA-ubi-K48
HA-ubi-K63
IP:TBK1
IB: TBK1
IB: TRIM28

Figure. S1

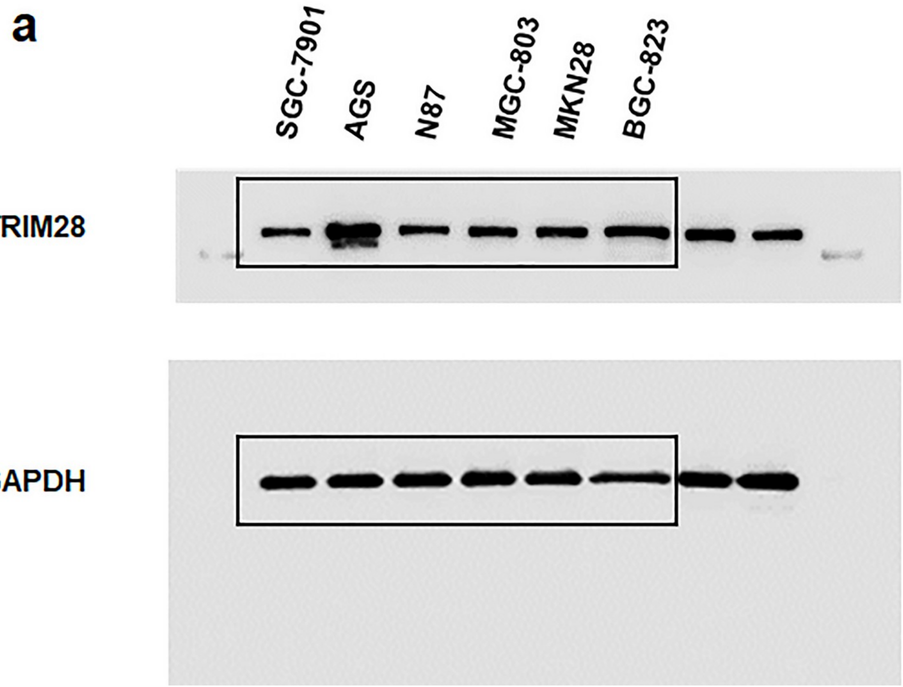

Figure. S2

c

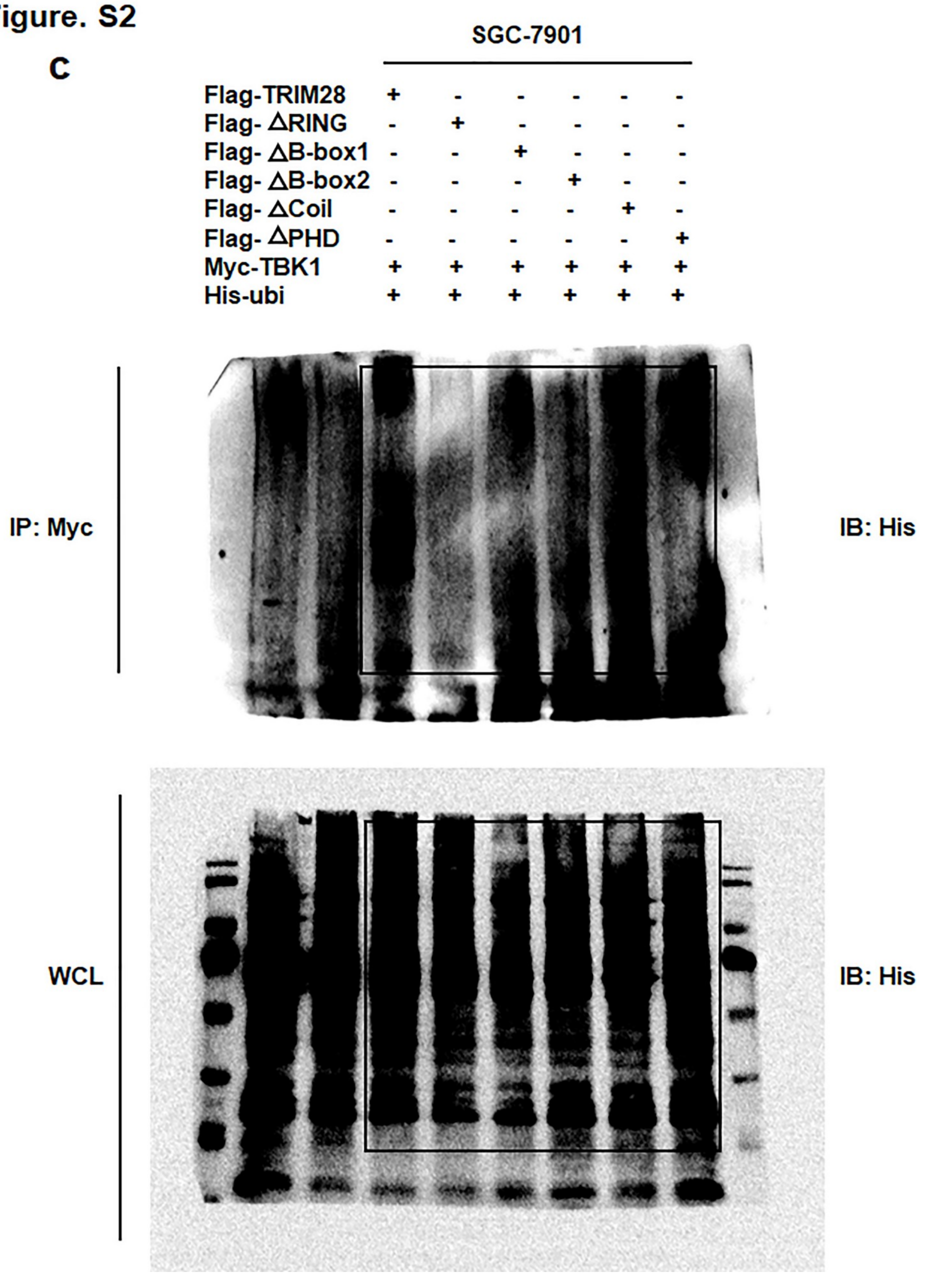
SGC-7901
Flag-TRIM28 + - - - - -
Flag- △RING - + - - - -
Flag- △B-box1 - - + - - -
Flag- △B-box2 - - - + - -
Flag- △Coil - - - - + -
Flag- △PHD - - - - - +
Myc-TBK1 + + + + + +
His-ubi + + + + + +
IP: Myc
IB: His
WCL
IB: His

Figure. S8

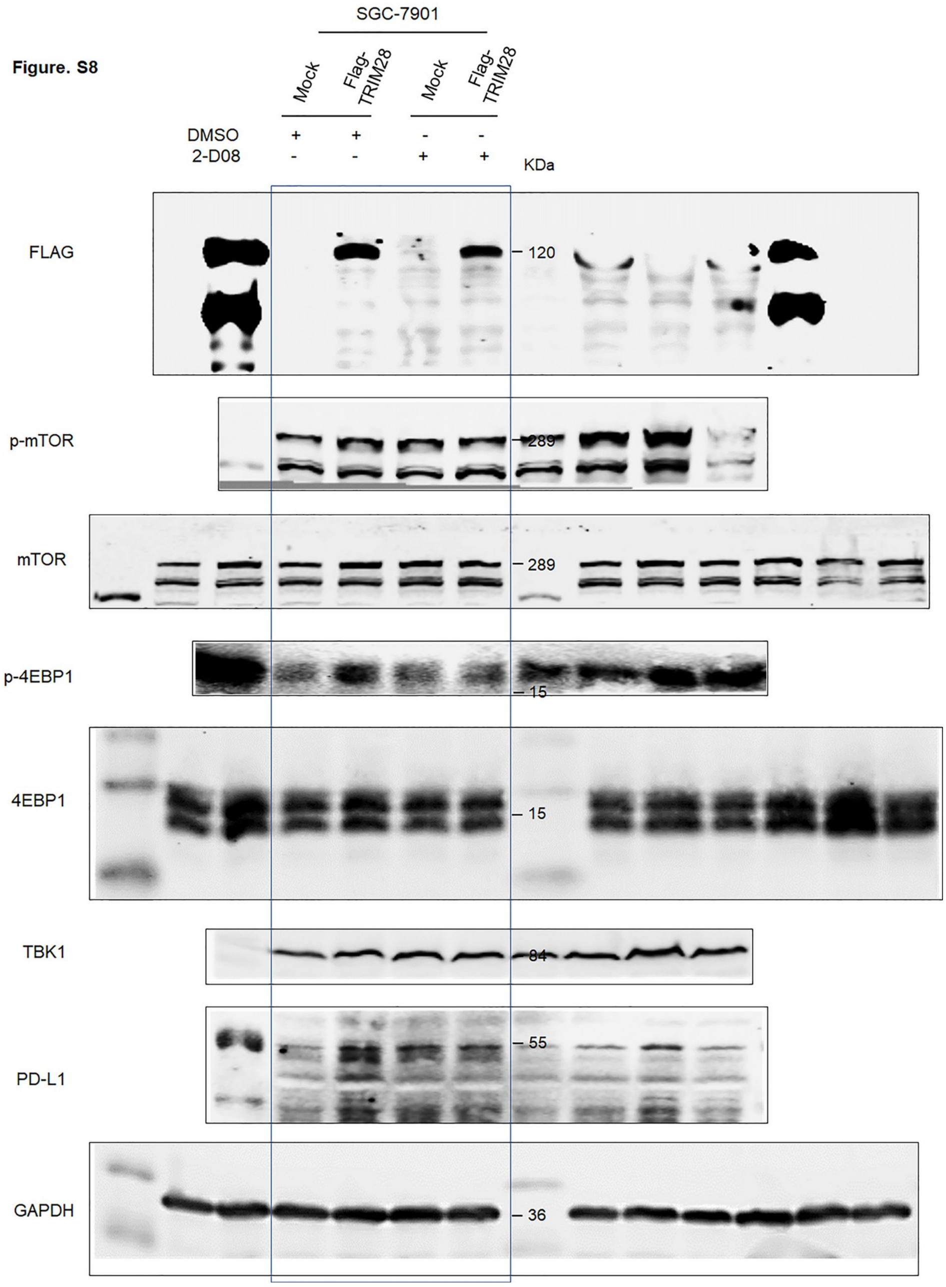
SGC-7901
Mock
Flag-TRIM28
Mock
Flag-TRIM28
DMSO + + - -
2-D08 - - + +
KDa
FLAG
120
p-mTOR
289
mTOR
289
p-4EBP1
15
4EBP1
15
TBK1
84
PD-L1
55
GAPDH
36
